# Immunotherapy associated central nervous system complications in primary brain tumors

**DOI:** 10.3389/fonc.2023.1124198

**Published:** 2023-02-16

**Authors:** Megan Mantica, Jan Drappatz

**Affiliations:** Department of Neurology, University of Pittsburgh, University of Pittsburgh Medical Center (UPMC), Pittsburgh, PA, United States

**Keywords:** immune checkpoint inhibitor, glioma, neurotoxicity, primary brain tumor, CNS, CAR T Cell, oncolytic viruses

## Abstract

Advances clarifying the genetics and function of the immune system within the central nervous system (CNS) and brain tumor microenvironment have led to increasing momentum and number of clinical trials using immunotherapy for primary brain tumors. While neurological complications of immunotherapy in extra-cranial malignancies is well described, the CNS toxicities of immunotherapy in patients with primary brain tumors with their own unique physiology and challenges are burgeoning. This review highlights the emerging and unique CNS complications associated with immunotherapy including checkpoint inhibitors, oncolytic viruses, adoptive cell transfer/chimeric antigen receptor (CAR) T cell and vaccines for primary brain tumors, as well as reviews modalities that have been currently employed or are undergoing investigation for treatment of such toxicities.

## Introduction

Primary brain tumors are tumors that originate within the brain, spinal cord, and spinal fluid. CNS tumors are relatively uncommon in the United States with 25,000 cases annually and represents 1.3% of all new cancer cases (1). Primary brain tumors are classified by histopathologic criteria and immunohistochemical (IHC) data according to the World Health Organization (WHO) Classification of Tumors of the CNS published in 2021 (2). CNS tumors are a diverse constellation of over one hundred different histological subtypes with distinct epidemiology, clinical characteristics, prognosis, and treatments (3). While more than half of all primary CNS tumors are benign, they can cause significant morbidity, and the more aggressive tumors have high mortality often with limited effective treatment options in the recurrent setting. Glioblastoma Multiforme (GBM) is the most common primary malignant brain tumor in adults and despite aggressive treatment with surgery, radiation (RT) and chemotherapy, prognosis remains poor with limited effective treatment options in the recurrent setting with an expected five-year survival rate of less than 5% (2).

Treatment of recurrent GBM and other high grade gliomas (HGG) outside of clinical trials often involves re-resection, re-irradiation with either short course fractionated radiation or single fraction stereotactic radiosurgery, temozolomide re-challenge, lomustine or bevacizumab (4). There have been multiple clinical trials combining therapeutic agents, but thus far, have shown no improvement in outcome (4). As such, there is an urgent need for novel therapeutic agents and combination regimens for treatment of recurrent HGG.

Given the robust response to immunotherapy observed in other cancer types, immunotherapy is an appealing treatment option for recurrent malignant CNS tumors including HGG and GBM. The emergence of immune checkpoint inhibitors (ICIs) for solid tumors and chimeric antigen receptor T (CAR T) cells for liquid tumors has led to substantial tumor response and improved survival multiple extra-cranial cancer types (5–7) and is increasingly used in patients with secondary brain metastases (8). However, neurologic toxicities associated with immunotherapy can limit the potential anti-tumor effects, can involve the entire neuroaxis leading to permanent discontinuation of therapy and can cause significant morbidity and even mortality (9). Diagnosing CNS toxicity in patients with primary brain tumors have unique and distinct challenges given the overlapping presentations of immune-related toxicity, tumor pseudoprogression and true tumor progression. Many symptoms such as fatigue, confusion, and headache are non-specific and difficult to classify into a specific neurologic syndrome and are often underrecognized and underreported and has not been studied systemically in patients with primary brain tumors. As a result, there is substantial variation in reporting of adverse events among publications and there may be a bias towards reporting severe events in the literature. Given the emergence of a substantial number of immunotherapy trials for primary CNS tumors, there is an urgent need to further understand the spectrum of immune-related neurological CNS toxicity that can occur and optimally manage these immune related adverse events. This review highlights the emerging and unique CNS complications associated with immunotherapy including checkpoint inhibitors, oncolytic viruses, adoptive cell transfer/chimeric antigen receptor (CAR) T cell and vaccines for primary brain tumors.

## Methods

### Objective

To evaluate the CNS complications associated with immunotherapy for primary brain tumors including checkpoint inhibitors, oncolytic viruses, adoptive cell transfer/chimeric antigen receptor (CAR) T cell and vaccines; and to prepare a review on the available evidence.

### Criteria for considering studies for this review

Types of studies: Randomized controlled trials (RCTs), quasi‐randomized trials, non‐randomized studies, and controlled before‐and‐after studies that include relevant concurrent comparison groups. We do not expect to find cluster‐randomized trials. In view of the limited number of trials in primary brain tumors, we included case‐control studies, studies without a control group, case series and case reports. Studies included a minimum of 1 participant.

Types of participants: People aged 6 months of age and older diagnosed with a primary brain tumor according to 2021 WHO Classification of Tumors of the Central Nervous System. Participants with new diagnosis, first and subsequent recurrences were included.

Types of interventions: Any active immunotherapy treatment (checkpoint inhibitors, oncolytic viruses, adoptive cell transfer/CAR T cell and vaccines) or treatment combination compared with another active treatment (surgical resection, radiation, chemotherapy).

### Primary outcomes

Severe adverse events (grade 3 or higher according to a standardized measurement tool, such as the Common Terminology Criteria for Adverse Events (CTCAE) v 5.0)

Incidence of pseudoprogression (according to a standardized measurement tool, such as Response Assessment in Neuro-Oncology (RANO) or Immunotherapy Response Assessment in Neuro-Oncology (iRANO)).

### Search methods for identification of studies

Electronic searches: For evidence on the safety and incidence of serious adverse events in the CNS, we will prepare the search strategies and conduct the searches of the following databases from 1990 onwards. We will use Cochrane Central Register of Controlled Trials (CENTRAL), in the Cochrane Library; MEDLINE Ovid (from 1990 onwards); Embase Ovid (from 1990 onwards), and PubMed (from 1990 onwards).

## Adoptive cell transfer/CAR-T cell

Adoptive cell transfer or T-cell therapy is a form of cancer immunotherapy involving the autologous or allogeneic transplant of tumor-infiltrating lymphocytes (TILs) or genetically modified T cells expressing novel T-cell receptors or chimeric antigen receptors (CAR-T cell therapy). Adoptive cell transfer has shown remarkable success in the treatment of hematologic malignancies and is a promising therapy for the treatment of other solid malignancies, including brain tumors. CAR-T cells are synthetically engineered cells that express a chimeric antigen receptor against specific tumor antigens without major histocompatibility complex (MHC) molecules or antigen presenting cells and then can individually activate multiple immune cells and secrete cytokines to promote effector function and cell trafficking allowing self-amplification and improved T cell response (10, 11). Although the first CAR-T cells were developed in 1987, progress and discoveries in their anti-tumor function have led to several generations of CAR T engineering now with fourth generation CAR T compositions termed ‘armored CAR’ or TRUCKs (T-cells Redirected towards Universal Cytokine Killing). TRUCKs are specific armored CAR T cells that modulate the tumor microenvironment by secreting cytokines to interfere with the immunosuppressive cytokine profile to increase anti-tumor activity of the CAR T cells and tissue-resident immune cells (12). CAR T cells have been shown to be highly efficacious against chronic lymphoblastic leukemia (CLL), acute lymphoblastic leukemia (ALL) and diffuse large B cell lymphoma (DLBCL) leading to FDA approval of two CD19 antigen-specific CAR T cell products, tisagenlecleucel (CTL019, Kymriah^©^) and axicabtagene ciloleucel (Yescarta^©^), for the treatment of refractory B cell hematologic malignancies (13). Given the promising results in extracranial malignancies, the use of adoptive cell transfer is emerging as a potential therapeutic option for patients with brain cancers.

Single antigen CAR T cell therapies are under investigation in malignant brain tumors, including glioblastoma multiforme (GBM), the most common malignant brain tumor in adults, medulloblastoma, ependymoma and diffuse intrinsic pontine glioma in pediatric patients. Several molecules have been identified as potential tumor antigens for CAR T cell therapy targeting primary brain tumors through immunohistochemical (IHC) analysis including EGFR/EGFRvIII, IL13Rα2, HER2 and B7-H3 (14). Ideally, tumor antigen targets should be expressed on all cancer cells within a primary tumor, but minimally expressed on normal tissues to avoid killing of normal cells by the CAR T cells. Non-specific targeting can lead to toxicity and cytokine release syndrome (CRS), which is a potentially significant and well described side effect of CAR T cell therapy.

Neurotoxicity is one of the most common and potentially fatal complications of CAR T cell therapy with an incidence ranging from 2% to as high as 60%–70% (7, 15, 16). Symptoms range from mild delirium, language dysfunction to seizures, coma, and fulminant intracerebral edema (17, 18). This syndrome of neurological adverse events is designated “immune effector cell-associated neurotoxicity syndrome” (ICANS) by the American society of Transplantation and Cellular Therapy. The variability in incidence noted in hematologic malignancies is attributed to the incidence of CRS, type of malignancy, properties of individual CAR constructs and differences in grading schemes. Unlike hematologic malignancies in which there is expression of a single tumor antigen, solid tumors have significant antigen heterogeneity and many antigens associated with solid tumors are also expressed on healthy tissue, increasing the risk of off-target toxicity. In solid tumor malignancies, CRS is less well defined and incidence of ICANS is not well reported with setback in June of 2021 for the use of CAR T in solid tumors after Tmunity Therapeutics halted its phase 1 clinical trial of prostate-specific membrane antigen (PSMA)-directed, TGF- β sensitive CAR T cells for prostate cancer after two fatalities from ICANS.

The presence of baseline neurological disorders or magnetic radiographic imaging (MRI) changes is reported to increase the risk of ICANS and has led to a cautious use of CAR T in patients with known CNS involvement of leukemia or lymphoma (19, 20) and presents potential additional risk factor in patients with primary brain tumors, whom at baseline often have neurological deficits and distinguishing between ICANS and tumor progression, tumor pseudoprogression or non-specific neurological symptoms such as headache, confusion, fatigue and altered mental status presents a unique challenge. Expanding on this specific and unique toxicity profile in patients with primary brain tumors, neurological symptoms related to CAR T cell mediated inflammation in sites of CNS disease has been termed by Majzner et al., 2022 as ‘tumor inflammation-associated neurotoxicity’ (TIAN). They describe two categories if TIAN: the first category of TIAN relates to increased intracranial pressure (ICP) intracranial space constraints secondary to inflammation-induced tissue edema and/or obstruction of CSF flow; the second category of TIAN relates to primary dysfunction of brain or spinal cord structures affected by inflammation manifested as transient worsening or recrudescence of pre-existing symptoms. The authors propose TIAN as an emerging classification of ICANS for tumors in the CNS (21). Adopting this terminology, in this review we will refer to CNS-associated CAR-T neurological complications as TIAN.

In 2015, Brown et al. performed a first in human pilot study assessing the feasibility, safety, and toxicity of intracranial administration of first-generation autologous interleukin-13 receptor subunit alpha-2 (IL13Rα2)-specific CAR CD8+ T cell clones for the treatment of recurrent high-grade glioma (WHO grade III and IV) following tumor resection. The IL13 Rα2 is a monomeric receptor for interleukin 13 that is present in up to 60% of GBMs and is associated with the activation of proinflammatory and immune pathways (22). Overexpression of IL13 Rα2 activates the phosphatidylinositol-3 kinase/AKT/mammalian target of rapamycin pathway, leading to poor prognosis and increased tumor invasiveness in GBM (23). Intracranial delivery of the IL13-zetakine+ CTL clones into the resection cavity of three patients with recurrent high-grade glioma (HGG) *via* Rickham catheter placement was well-tolerated with all three patients reporting grade 3 headaches and one patient with shuffling gait and tongue deviation attributed to neurologic toxicity from CAR T requiring hospital admission and treatment with single infusion of 10mg intravenous dexamethasone. All three patients were noted to have increase in gadolinium contrast enhancement on MRI immediately following CAR T administration and 2/3 had decreased contrast enhancement on follow-up MRI after a few months (24). This established the feasibility and safety of locally delivered autologous CAR T cells without development of serious treatment-associated side effects.

Following this pilot trial, Brown and colleagues 2016 modified the IL13Rα2-targeted CAR T cells by incorporating 4-1BB (CD137) co-stimulation and a mutated IgG4-Fc linker to improve antitumor potency, improve T-cell persistence and reduce off-target Fc-receptor interactions. They report on a single patient with multifocal recurrent glioblastoma with leptomeningeal disease with five intracranial lesions and spinal cord drop metastases that showed evidence of a complete response according to Response Assessment in Neuro-Oncology criteria after 16 cycles of treatment with six intracavitary infusions and 10 intraventricular infusions. Infusions were not associated with Grade 3 or higher toxicity. Grade 1 and 2 events were observed within 72 hours after CAR T cell infusions including headaches, generalized fatigue, myalgia, and olfactory auras (25). However, the use of concurrent dexamethasone dosed upwards of 4mg daily may have influenced the reported safety profile. This study further corroborated the safety and feasibility of intracavitary and intraventricular delivery routes.

Epidermal growth factor receptor variant III (EGFRvIII) targeting CAR T cells are also being tested in patients with recurrent GBM. EGFRvIII results from an in-frame deletion of exons 2–7 of the EGFR gene and is the most common mutation of this receptor that occurs in upwards of 30% of GBM patients (26). In a pivotal phase 1 clinical trial by O’Rourke et al., 2017 (16), the most common CNS neurologic adverse events included dysgeusia, headaches, cranial neuropathies (facial and hypoglossal nerve weakness), lethargy, dysarthria, and dizziness. Three participants experienced clinically significant neurologic adverse events associated with EGFRvIII CAR T infusion with grade 4 cerebral edema, grade 3 seizure and altered mental status and grade 3 intracerebral hemorrhage. For intracerebral edema and seizure, siltuxumab (anti-IL-6) and high dose steroids were administered for treatment to treat the hypothesized intracranial cytokine release (27). Likewise, Goff et al., 2019 evaluated the use of IV administration of EGFRvIII CAR T therapy in 18 patients with recurrent glioblastoma. No neurological DLTs were noted, but evidence of grade 2 neurologic symptoms or suspected seizure activity was observed in 10/18 patients resulting in corticosteroid adjustment in four and antiseizure medication adjustment in three participants (28).

B7-H3 (CD276) is a type I transmembrane protein that plays a critical role in the activation or inhibition of T-cell function, is highly overexpressed in a wide range of cancers and correlates with negative clinical outcomes and poor prognosis. Tang et al., 2021 noted transient disease regression of a single patient with recurrent GBM after administration of B7-H3 targeted CAR-T (29). They reported grade 2 headaches associated with intracavitary administration of CAR-T.

Human epidermal growth factor 2 (HER2) is a transmembrane glycoprotein that is expressed in several CNS tumors including GBM, ependymoma, and medulloblastoma, but no expressed in normal brain tissue, making it an appealing target for immunotherapy given on-target specificity with limited off-target potential toxicity (30). Ahmed et al., 2017 evaluated the safety and feasibility and the anti-glioblastoma activity of IV HER2 CAR T therapy in recurrent pediatric and adult HER2-positive glioblastoma. 17 patients were evaluated (10 adult patients and 7 pediatric patients with rage 10-69 years). The authors report that infusions were well tolerated and no neurologic DLTs observed. No grade 3 or 4 neurological toxicities were reported and only three grade 2 adverse neurological events attributed to CAR T infusion were observed with two grade 2 seizures and one grade 2 headache (31). Use of corticosteroids and/or antiseizure medications was not clarified.

The most recent study to date by Majzner et al., 2022 is a phase 1 dose-escalation trial of autologous GD2-CAR T cells in children and young adults with pontine and spinal cord H3K27-mutated diffuse midline gliomas (21). This study evaluated the safety and tolerability and identified the recommended phase II dose of engineered disialoganglioside GD-2 CAR T cell infusion. They describe four patients who underwent first-in-human treatment with intravenous and intraventricular GD-2 CAR T cells. There were significant associated and anticipated neurological toxicity termed TIAN including cranial nerve symptoms, elevated ICP with hemiplegia, extensor posturing, and clinically significant cerebral edema, headache, hydrocephalus, and transient worsening of baseline neurological symptoms. Increased ICP was reported in 2/4 patients and requires CSF drainage from Ommaya reservoir followed by administration of anakinra (IL-1R antagonist) and corticosteroids. Transient worsening of baseline neurological symptoms was treated with anakinra, tocilizumab and/or corticosteroids with improvement back to baseline neurological examination (21). Given the location of diffuse midline gliomas involvement in the brainstem, anticipated development of neurological symptoms with a predetermined treatment algorithm was conducted and included placement of Ommaya reservoir to monitor ICP, removal of CSF *via* Ommaya reservoir, hypertonic saline, anti-cytokine agents (anakinra and tocilizumab) and corticosteroids. The prompt recognition and early treatment to mitigate neurologic toxicity enabled the safe and feasible delivery of intravenous and intraventricular administration of GD-2 CAR T therapy. This study highlighted the main categories of TIAN as it related to ICP and inflammation-induced cerebral edema and transient recrudescence of baseline neurological symptoms and underscored the need for clear and timely management algorithms to mitigate potentially fatal treatment associated toxicity.

In hematologic malignancies, treatment of CRS often targets elevated cytokines (most elevated cytokines typically seen are IL-10, IL-6 and INF- γ) and is managed using corticosteroids interleukin-6 blockade (32, 33). IL-6 blockade *via* tocilizumab or siltuximab have shown dramatic reversal of severe CRS in patients treated with CART-19 and is actively being studied in other therapies (34, 35). However, there is some controversy regarding the use of Tocilizumab for ICANS in absence of CRS given poor blood brain barrier penetration and potential shunting of IL-6 to the CSF and worsening ICANS. In the Zuma-1 trial (cohort 3), patients were treated with prophylactic tocilizumab in combination with CART19 resulting in reduction in severe CRS, but there was a trend toward increased ICANS rates and severity thought to be due to peripheral IL-6 receptor blockade resulting in shunting of IL-6 to the CSF space and worsening CNS toxicity (36, 37). The use of IL-6 blockade for treatment of CAR T associated neurotoxicity in primary brain tumors is not well described with paucity of literature demonstrating efficacy in improvement in neurological symptoms after IL-6 blockade. In hematologic malignancies, the standard approach for treatment of ICANS includes supportive care and administration of corticosteroids. Novel approaches to inhibit inflammatory cytokines are being investigated including use of IL-6 inhibitor, siltuximab (38), Il-1 inhibitor, anakinra (39), and granulocyte-macrophage colony-stimulating factor neutralization with lenzilumab (40) and endothelial protection using debfibrotide (41).

Another potential method of managing TIAN is incorporation of safety switches either by inclusion of transgenic enzymes selectively activated by a cytotoxic pro-drug known as suicide genes such has herpes simplex virus-thymidine kinase or inducible caspase 9 or by expression of surface molecules such as CD20 or EGFR that can be targeted using clinically approved monoclonal antibodies (42–44). Suicide genes engineered into the CAR T cells provide a switch to induce apoptosis of CAR T cells to prevent potentially toxic immune stimulation and reverse CRS (44, 45). While suicide gene iCasp9 was incorporated into the GD-2 CAR T construct, the engineered suicide switch was not utilized given absence of life-threatening toxicity that was refractory to IL-6 blockade and corticosteroids (21).

## Vaccine therapy

Vaccine therapy for primary brain tumors is based on the tumor-specific response to the introduction of foreign antigens to antigen presenting cells to induce and enhance the immune system to eradicate the tumor. Antigen targets for vaccines are classified into two broad categories: 1) tumor-associated, which are over-expressed in tumors such as survivin and Wilms tumor 1 in GBM, or 2) tumor-specific, which are exclusively expressed by tumor cells such as EGFRvIII and isocitrate dehydrogenase (IDH) R132H in GBM and astrocytoma grade 4 (46, 47). Another vaccine target is neoantigens, which are proteins that arise from mutations within tumor cell and typically vary between cells and between individuals. Neoantigen vaccines utilize personalized sequencing data from whole exome and RNA of individual patient’s tumor to identify specific individual mutations (48). Many potential tumor antigens do not originate from mutations but are the result of overexpression of normal proteins. This is the case with tumor-associated antigens that are also expressed in other tissues and targeting the antigen may lead to off-target effects and toxicity (49).

Broadly, the basis of primary brain tumor vaccine therapy starts with two main vaccine platforms including peptide vaccines and nucleic acid vaccines (DNA or RNA) that are then packaged into a vehicle including either dendritic cells, viral vectors, heat shock proteins, or montanide, then paired with an adjuvant such as tetanus toxoid, poly-ICLC, imiquimod, GM-CSF, or immune-checkpoint inhibitors to boost the efficacy of the vaccine (50). Vaccines are given intra-nodally, intramuscularly, intra-dermally or intravenously and are presented by antigen presenting cells (APCs) to T cells in the lymph node then primed T cells migrate to the tumor site where they mount an anti-tumor response (50). To date, there are over 150 clinical trials incorporating vaccine therapy for treatment of gliomas. Given the considerable number of clinical trials in primary brain tumors utilizing vaccine therapy, highlighting the potential CNS toxicity of such treatments is imperative for ongoing safe and novel vaccine therapy approaches in primary brain tumors. Vaccine therapy in CNS tumors has been developed over the last 30 years (**Table 1**) with the first vaccine trial in 1993; yet, to date, there have only been three vaccines that have reached phase III clinical trials: Rindopepimut, DCvax, and PPV (51–53).

**Table 1:** CNS Toxicity of Primary Brain Tumor CAR-T Trials.

Clinical Trial	NCT	Primary Brain Tumor	Tumor Antigen Target	Number of Patients	Method of Delivery	Neurologic Dose-Limiting Toxicity	CNS Adverse Events	Pseudoprogression
Brown et al. 2015 (24)	NCT00730613	rHGG	IL13Rα2	3	Intracavitary	Headache (1/3)	Grade 3 Headache Grade 3 Shuffling gait and tongue deviation Grade 3 Leukopenia, headache, and fatigue	3/3
Brown et al. 2016 (25)	NCT02208362	rGBM, multifocal	IL13Rα2	1	Intracavitary and Intraventricular	None	Grade 2 Headaches, generalized fatigue, myalgia, and olfactory auras	0/1
O’Rourke et al. 2017 (27)	NCT02209376	rGBM, multifocal	EGFRvIII	10	IV	None	Grade 4 Cerebral edema Grade 3 Seizure and altered mental status Grade 3 Intracerebral hemorrhage	Not Reported
Ahmed et al. 2017 (31)	NCT01109095	rGBM	HER2	17	IV	None	Grade 2 seizures (2/17) Grade 2 headaches (1/17)	Not Reported
Goif et al. 2019 (28)	NCT01454596	rGBM	EGFRvIII	18	IV	None	Grade 3 motor weakness and urinary incontinence Grade 2 neurologic symptoms or seizure (10/18)	Not Reported
Tang et al. 2021 (29)	NCT03241940	rGBM	B7-H3	1	Intracavitary	None	Grade 2 headache	Not Reported
Majzner et al. 2022 (21)	NCT04196413	H3K27M-mutated DMG	GD-2	4	IV Intraventricular	None	Increased ICP Hydrocephalus Worsening baseline neurological symptoms Headache	1/4

CNS, central nervous system; DMG, diffuse midline glioma; IV, intravenous; r- recurrent; HGG, high grade glioma; GBM, glioblastoma.

Peptide vaccines are short chain 20-30 amino acids sequences that are synthesized to form an immunogenic peptide molecule representing a specific epitope of an antigen in order to induce activation of T cells (54). In GBM, peptide vaccines are some of the most commonly used vaccine platforms. EGFRvIII, CMV pp65, TERT, IDH1, surviving, WT1 have been used as single-antigen peptide and include epitopes of tumor-associated or GBM-specific antigens (55, 56). The seminal phase III clinical trial ACT IV investigating the use of Rindopepimut, a peptide vaccine targeting the EGFR deletion mutation EGFRvIII, plus temozolomide in newly diagnosed GBM in its failure to improve OS highlighted the limitation of single-antigen approach due to tumor heterogeneity and immune selection (51). Importantly, this trial also demonstrated the safety and tolerability of peptide vaccines as it related to CNS toxicity. The most common grade 3-4 CNS adverse events compared to control included brain edema (2% vs 3%), seizure (2% vs 2%), and headache (2% vs 3%). There was no evidence for increased toxicity that might theoretically arise due to Rindopepimut-induced immune infiltration of the brain such as cerebral edema or seizure (51). Similar CNS adverse events were reported in the phase II ReACT trial of Rindopepimut plus bevacizumab, again without evidence for increased CNS toxicity related to the peptide vaccine (57). Another pivotal advance in vaccine therapy for primary brain tumors is the use of cytomegalovirus (CMV) phosphoprotein 65 (pp65), which is tumor-specific and not present on normal brain tissue (58, 59). Preliminary results of the Phase I trial PRiME (NCT03299309) which is testing the peptide vaccine PEP-CMV in malignant glioma and

medulloblastoma patients has demonstrated no grade 3 or 4 toxicities related to the vaccine and thus far no report of CNS toxicity attributable to the vaccine (59).

Another tumor-specific single antigen vaccine that has been studied is IDH1 (55, 60). Eighty percent of low-grade gliomas have an IDH1 mutation, of which IDH1 R132H substitution is the most common. Three phase I clinical trials have studied peptide vaccines targeting IDH1R132H including NOA-16 (NCT02454634), RESIST (NCT02193347) and AMPLIFY-NEOVAC (NCT03893903) and one trial that is pending ViCToRy (NCT05609994). NOA-16 evaluated patients with grade III or grade IV IDH1R132H mutation astrocytoma and the RESIST trial evaluated patients with grade II astrocytomas. Pseudoprogression in peptide vaccine arm versus control was 37.5% vs 16.7% without apparent association with age, extent of resection, standard of care treatment or WHO grade. In NOA16, pseudoprogression was associated with the onset of peripheral IDH1-vaccine-induced immune responses and was restricted to patients with transient or sustained T cell immune responses (60, 61). Additional tumor-associated antigens have been tested including survivin (phase II study of SurVaxM vaccine in newly diagnosed GBM: NCT02455557) and WT1 (multiple clinical trials underway using DSP-7888 for pediatric HGGs and progressive GBM) (62). Final data from the phase 2a single-arm trial of SurVaxM for newly diagnosed glioblastoma evaluated 63 patients with newly diagnosed GBM with no serious adverse events (63).

A major challenge with single agent peptide vaccines is the potential for tumor immune escape. As such, clinical trials have investigated multiple agent peptide vaccine targets. A trial by Pollack et al., 2014 used glioma-associated antigen (GAA) vaccine consisting of EphA2, interleukin-13 receptor alpha 2 (IL-13Rα2), and survivin in HLA-A2–positive children with newly diagnosed brainstem glioma and HGG. Twenty-six children were enrolled, 14 with newly diagnosed BSG treated with irradiation and 12 with newly diagnosed BSG or HGG treated with irradiation and concurrent chemotherapy. Five children (19%) had symptomatic pseudoprogression, which responded to dexamethasone and was associated with prolonged survival. Two children developed acute neurological worsening several months after vaccination, one with central hyperventilation and one with multiple cranial neuropathies resulting in aspiration pneumonia and necessitating intubation. MR imaging demonstrated increased tumor size and enhancement during vaccination followed by radiographic stabilization. Four of five children with BSG and pseudoprogression survived at least 18 months after diagnosis versus two of 15 without pseudoprogression (64). Other early phase I and I/II clinical trials investigating multiple antigen targets using IMA950, a combination of 11 tumor-associated antigens, in newly diagnosed GBM and IMA950/polyinosinic-polycytidylic acid stabilized with polylysine and carboxymethylcellulose (ICLC) in newly diagnosed high grade astrocytomas. Both trials demonstrated that multi-antigen peptide vaccines were safe and well tolerated. However, the most common SAE reported was seizures 8/39 (20%) and 9/19 (47%), respectively (65, 66). Pseudoprogression was observed in 4/19 (22%) in the IMA950/poly-ICLC trial and observed most frequently after the fourth vaccination (65). Pseudoprogression was treated with high dose steroids followed by a steroid taper over 10 days with radiographic improvement in cerebral edema with minimal tumor progression. It was felt that there was no association between residual tumor volume and extent of cerebral edema, and the occurrence of increase edema was not thought to be associated with modification of the vaccine formulation (65).

Based on the current early phase clinical safety data utilizing peptide vaccines either single-antigen or multi-antigen peptide targets, the most common CNS toxicities are cerebral edema or pseudoprogression, seizures and headaches. Although there are clearly a limited number of clinical trials and small samples sizes for comparison, the percentage of participants within the trials that develop pseudoprogression appears similar between single-antigen and multi-antigen peptide vaccines. More studies are needed to compare the rates of pseudoprogression and any association with the number of antigen targets presented.

Another common vaccine platform used in primary brain tumors are nucleic acids (DNA or RNA). DNA-based vaccines are currently being studied in clinical trials in GBM (NCT03491683, NCT04015700, NCT02718443). This platform utilizes the method of encoding tumor-associated antigens and immune-stimulating cytokines into bacterial DNA plasmids that are then inserted into host cells resulting in presentation on both MHC Class I and II molecules to activate innate immune response (67). The interim analysis for the phase I/II study using DNA vaccines INO-5401 and INO-9012 combined with PD-1 antagonist cemiplimab in newly diagnosed GBM thus far has reported tumor inflammation (7.7%) and seizures (7.7%) as the second and third most common adverse event following thrombocytopenia (11.5%) (68). RNA-based vaccines are actively being investigated, but to date, no clinical data is available pertaining to CNS toxicity of RNA nucleic acid vaccine platform.

One of the most used vaccine vehicles is dendritic cells (DCs). DCs are antigen presenting cells (APCs) that traffic *via* the tumor draining lymph nodes of the brain to the deep cervical lymph nodes, capture and present exogenous antigens *via* MHC Class I molecules to stimulate CD8+ T cells and induce an adaptive and innate immune response. As such, DCs are an ideal vehicle for vaccines in CNS tumors (69). DC vaccines are derived from patient-derived DCs from peripheral blood cultured ex vivo with pro-inflammatory cytokines such as IL1beta, TNF-alpha and PGE1 (70). They are then pulsed with antigens including peptides, tumor RNA, tumor-associated antigens, tumor-derived exomes, and tumor lysates (70). DC vaccine studies in CNS tumors have explored multiple types of loaded antigens including various tumor-associated, tumor-specific, and neo-antigen peptides and nucleic acids. There are four main categories of DC vaccines: single tumor antigens, multiple antigens, autologous whole-tumor lysate, and glioma stem cells (50). Over 30 clinical DC vaccines clinical trials have been performed between 2001 and 2022 in primary brain tumors across these broad categories with majority of trials reporting minimal to no neurologic toxicity (**Table 1**).

Single tumor antigen dendritic cell vaccines use single antigen targets such as EGFRvIII packaged into a dendritic cell vehicle. One of the early studies evaluating the use of tumor-specific DCs vaccines targeting EGFRvIII in glioblastoma is the phase I trial by Sampson et al., 2010. Patients underwent leukapheresis to obtain peripheral blood mononuclear cells (PBMC) for DC generation and pulsed with PEPvIII that spans the fusion junction of EGFRvIII conjugated to keyhole limpet hemocyanin and bathed with cytokines TNF-alpha and IL-6. Twelve patients received three vaccines in equal doses two weeks apart then followed without additional therapy until radiographic or clinical progression. Toxicity was reported as minimal, and there were no adverse events exceeding grade 2 toxicity at any of the DC doses tested (71). There was no report of CNS neurotoxicity. Several other early phase I and I/II studies evaluating single tumor antigen pulsed DCs have similarly shown minimal overall toxicity and paucity of CNS neurotoxicity (**Table 1**) including a phase I trial for recurrent GBM I which patients received DCs pulsed with WT1 (72, 73) and a phase I trial of IL-13Ra2 pulsed DCs (74). Likewise, the use of DCs pulsed with mRNA encoding CMVpp65 antigen has been studied by Batich et al., 2017. Eleven patients with newly diagnosed GBM received dose-intensified temozolomide and three doses of CMVpp65 DC vaccine. No patients experienced neurologic AEs related to the pp65-DC vaccine (75). Batich et al. conducted three sequential clinical trials using CMVpp65 DC vaccines in patients with newly diagnosed GBM. Pooled results from the three separate trials demonstrated that nearly 1/3^rd^ of the GBM patient population receiving CMV-specific DC vaccines resulted in exceptional long-term survival and no reported CNS specific neurotoxicity (76).

One proposed benefit of DC vaccine use over other immunotherapy treatments such as adoptive cell transfer is that DC vaccines can be pulsed with multiple target antigens to generate an immune response to a variety of targets and potentially overcome innate limitations in immunotherapy due to the heterogeneity of high-grade brain tumors. However, as the number of multiple antigen targets increases, the theoretical risk for off-target toxicity remains a concern (70). Several trials utilizing multi-agent DC pulsed vaccines have been studied to date including (72, 77, 78). The largest multi-peptide pulsed DC vaccine study is the phase II study of ICT-107, an autologous DC vaccine targeting six antigens on both tumor and cancer stem cells including HLA-A1–restricted, melanoma-associated antigen-1 (MAGE-1) and antigen isolated from immunoselected melanoma-2 (AIM-2), and the HLA-A2–restricted, human EGFR-2 (HER2/neu), tyrosinase-related protein-2 (TRP-2), glycoprotein 100 (gp100), and IL13 receptor alpha 2 (IL13Rα2). The specific targeted antigens were selected given high expression in GBM tumors with all six expressed in 83% of tumors (79). Notably, the multi-agent DC pulsed vaccines had minimal neurological toxicity with two trials reporting no neurologic toxicity (72, 77) and a single trial demonstrating modest neurologic toxicity compared to placebo with reported Grade 2 headaches (2.5% vs 16.3%), convulsions (8.8% vs 14%), partial seizures (6.3% vs 2.3%), and hemiparesis (5% vs 4/7%) and four grade 3 convulsions (5% vs 2.3%) (78). It is unclear whether the reported adverse events are related to DC vaccination administration or clinical neurological complications secondary to underlying high grade brain tumors, especially given unremarkable rates of neurologic events compared to placebo. While there are limited number of clinical trials using multi-agent pulsed DC vaccines, to date, there does not appear to be an increased number of reported neurologic adverse events compared to single-agent pulsed DC vaccine administration.

Another commonly and extensively studied approach to DC vaccination is the use of autologous surgical specimen tumor lysate pulsed DC vaccines, which offers a personalized approach unique to a patients’ individual tumor profile and allows immune presentation of neo-antigens and tumor-associated antigens (70). Like multi-peptide pulsed DC vaccines, this approach confers a potential risk for aberrant or indiscriminate antigen presentation and off-target toxicity. However, the theoretical increase in CNS toxicity has not materialized in several clinical trials to date with over twenty phase I, II and III clinical trials from 2001-2020 showing minimal neurologic toxicity (**Table 1**). A recent study by Bota et al., 2022 of 57 patients treated with Aivita GBM vaccine (AV-GBM-1) noted neurologic AEs attributed to the DC vaccine including headache (37%), seizures (33%), focal neurological deficits (28%), fall (18%), dizziness (18%), cerebral edema (16%), and confused/forgetful (11%) (80). There were noted 55 SAEs in total with 32/55 consider CNS SAEs including: seizures (16), falls (7), focal weakness (6) and cerebral edema (3); and one patient discovered decreased at home after refusing to go to the hospital after a fall two days prior, with immediate cause of death unclear (80). The largest tumor-lysate DC vaccine trial is the ongoing phase III randomized, double-blinded, placebo-controlled clinical trial of autologous tumor lysate-pulsed DC vaccine (DCVax^®^-L) for newly diagnosed glioblastoma (79). The interim analysis of 331 intention to treat (ITT) participants showed 93 (28.1%) patients with grade 3 or 4 adverse events related to ‘nervous system disorders’, with report of only 3 patients (0.9%) with cerebral edema and 2 patients (0.6%) reported seizures. Of note, the 28% nervous system disorders observed was not further delineated in this interim report and it was noted that the rate of adverse events was comparable to standard of care alone (79).

An emerging DC vaccination approach within the last several years is the use of glioma stem cells or cancer stem cells (CSC) pulsed DC vaccines. CSCs in brain tumors are defined by functional characteristics that include sustained self-renewal, persistent proliferation, and tumor initiation upon secondary transplantation (81). Two recent trials by Ogino et al., 2022 and Hu et al., 2022 tested the use of DCs pulsed with CSC for the treatment of low-grade gliomas and newly diagnosed and recurrent GBM, respectively. Hu et al., 2022 reported no neurologic toxicity associated with CSC DC vaccination (82) and Ogino et al. (83) reported occurrence of headaches (n=16), dizziness (n=3) and seizures (n=1) with multiple events in same participants, although no grade was assigned (82, 83). Overall, this approach is similar to tumor lysate pulsed DC vaccine and thus far shows a modest safety profile.

## Checkpoint inhibitors

Checkpoint inhibitors have been extensively studied in GBM treatment given their promising results in other solid malignancies. Through blocking of the tumor’s PD-L1 binding sites to T-cells, checkpoint inhibitors allow for T-cell activation, immune surveillance, and tumor recognition. Conversely, T-cells also have ability to induce adverse autoimmune-mediated complications (84). In addition to directly tumor associated complications such as cerebral edema, headaches or increased neurologic deficits, stimulation of autoantibody production can lead to cross-reactivity with antigens found on normal brain and nerve tissue (84).

Checkpoint inhibitor induced autoimmunity includes a wide spectrum of illnesses including hypophysitis, CNS vasculitis, meningoencephalitis, myositis, retinopathy, posterior reversible encephalopathy, myasthenia gravis, cerebellar degeneration, neuropathy, polyradiculopathy, autoimmune encephalitis, and progression of multiple sclerosis and has been described elsewhere (84). However, the incidence of checkpoint inhibitor induced central neurotoxicity in trials of patients with primary brain tumors has been low.

Several prospective trials have evaluated checkpoint inhibitors in patients with glioblastoma alone or in combination with standard therapy, i.e., bevacizumab in the relapsed setting or radiation and temozolomide for newly diagnosed glioblastoma (**Table 2**). Reported efficacy in all published phase 2 and 3 studies has been low, except for patients with mismatch repair deficiency (126) and in the context of small studies evaluating neoadjuvant PD-1 inhibitor use (summarized in **Table 2**). For example, a study by Cloughesy et al. randomized recurrent glioblastoma patients undergoing surgery to a single dose of neoadjuvant pembrolizumab versus no neoadjuvant dose prior to surgery, followed by adjuvant pembrolizumab in both arms (127), demonstrating an improvement in progression free survival (3.3 versus 2.4 months) and overall survival (13.7 versus 7.5 months) in patients who received pembrolizumab neoadjuvantly. The trial also demonstrated induction of TIL functional activation and production of an interferon (IFN)-γ response within the tumor. Headache and muscle weakness were the only treatment related neurologic toxicities in 47% and 50% of patients. Similar toxicities are commonly reported treatment-related adverse events in patients with central nervous system tumors; in fact, several of these adverse events were deemed unlikely to be related to the study drug but were included for completeness of data reporting. Other surgical window of opportunity trials are summarized in **Table 3**.

**Table 2:** CNS Toxicity of Primary Brain Tumor Vaccine Trials.

Clinical Trial	NCT	Phase	Primary Brain Tumor	Vaccine Target	Number of Patients	CNS Neurologic Adverse Events
Black et al. 1993 (85)	NCT	I	nAA, nGBM	ImuVert	15	None reported
Sampson et al. 2009 (86)	NCT	I	nGBM	EGFRvIII PEP-3-KLH	12	None reported
Sampson et al. 2010 (87)	NCT	II	nGBM	PEP-3-KLH	18	Grade 1 leukoencephalopathy
Sampson et al. 2011 (88)	NCT	II	nGBM	PEP-3-KLH	22	None reported
Schuster et al. 2015 (89)	NCT00458601	II	nGBM	PEP-3-KLH	65	None reported
Reardon et al. 2015 (90)	NCT	II	rGBM	PEP-3-KLH	36	Grade 3 back pain (6%) Gade 3 convulsion (11%) Grade 3 fall (3%) Grade 3 headache (3%)
Weller et al. 2017 (51)	NCT01480479	III	nGBM	EGFRvIII PEP-3-KLH	371	Headache (≥20%) Cerebral edema (5%)
Crane et al. 2013 (91)	NCT00293423	I	rGBM	HSPCC-96	12	None reported
Bloch et al. 2014 (92)	NCT00293423	II	rGBM	HSPCC-96	41	None reported
Fenstermaker et al. 2016 (93)	NCT01250470	I	rAA, rGBM	SurVaxM	9	None reported
Rosenfeld et al. 2010 (94)	NCT00262730	II	nGBM	Poly-ICLC	97	None reported
Rampling et al. 2016 (66)	NCT01222221	I	nGBM	IMA950	45	Grade 1 headache (n=20) Grade 2 headache (n=2) Grade 1 seizure (n=4) Grade 2 seizure (n=4) Grade 3 seizure (n=3) Grade 4 seizure (n=2)
Wheeler et al. 2008 (95)	NCT	I	GBM	Peptide	7	None reported
Hilf et al. 2019 (96)	NCT02149225	I	GBM	Peptide	15	Grade 3 Brain Edema
Keskin et l. 2019 (97)	NCT02287428	I	GBM	Peptide	10	None reported
Narita et al. 2019 (52)	NCT	III	rGBM	Peptide	58	Headache (n=2) Photophobia (n=1) Symptomatic epilepsy (n=1) Heaviness of head (n=1) Numbness of right ear (n=1) Dizziness (n=1) Dysguesia (n=1)
Ishikawa et al. 2007 (98)	NCT	I	GBM	Formalin-fixed Vaccine	12	None reported
Ishikawa et al. 2014 (99)	NCT	I.II	GBM	Formalin-fixed Vaccine	24	None reported
Kikuchi et al. 2001 (100)	NCT	I	AA, AO, GBM	Cultured glioma cells from surgical specimen DCs Vaccine	8	Seizure (n=1)
Yamanaka et al. 2003 (101)	NCT	I/II	AG, GBM	Tumor lysate from surgical specimen DCs Vaccine	10	Headache (n=1)
Yu et al. 2004 (87)	NCT	I	AA, GBM	Tumor lysate from surgical specimen DCs Vaccine	10	Grade 2 Seizures (25%) Grade 2 Headache (38%)
Rutkowski et al. 2004 (102)	NCT	I	PXA, GBM	Tumor lysate from surgical specimen DCs Vaccine	10	Grade 4 Cerebral Edema (n=1) Chemical meningitis (n=1)
Okada et al. 2007 (103)	NCT	I	AA, GBM	IL-4 gene , Tumor lysate from surgical specimen DCs Vaccine	7	Headache (n=1)
Caruso et al. 2004 (104)	NCT	I	AA, PXA, EPM, GBM	Tumor RNA from surgical specimen DCs Vaccine	7	None reported
Liau et al. 2005 (105)	NCT	I	GBM	Acid-eluted tumor-associated peptides DCs Vaccine	12	Seizure (n=1), Headache (n=2)
De Vleeschouwer et al. 2008 (106)	NCT	I/II	GBM	Tumor lysate from surgical specimen DCs Vaccine	56	Headache (n=9) Chemical Meningitis (n=1) Grade 4 Cerebral Edema (n=1) Transient focal neurological deficits (n=6) Seizures (n=4)
Walker et al. 2008 (107)	NCT	I	AA, GBM	Irradiated tumor cells DCs Vaccine	13	None reported
Wheeler et al. 2008 (95)	NCT	II	GBM	Tumor lysate from surgical specimen DCs Vaccine	34	None reported
Ardon et al. 2010 (108)	NCT	I	GBM	Tumor lysate from surgical specimen DCs Vaccine	8	Grade 4 status epilepticus (n=1) Grade 4 ischemic stroke (n=1) Grade 3 Seizures (n=1) Dysphasia (n=3) Transient confusion (n=2)
Ardon et al. 2010 (109)	NCT	I	AA, AO, PXA, GBM, AGG, DIPG, ATRT, EPM	Tumor lysate from surgical specimen DCs Vaccine	43	Headache (n=5)
Chang et al. 2016 (110)	NCT00293423	I/II	GBM	Heat shocked and irradiated tumor cells DCs Vaccine	16	None reported
Okada et al. 2011 (111)	NCT00766753	I/II	GBM, AA, AO, AOA	GAA epitopes from synthetic peptides (IL-13Rα2, EphA2, gp100, YKL-40) DCs Vaccine	22	Headache (n=7)
Prins et al. 2011 (112)	NCT00068510	I	GBM	Tumor lysate from surgical specimen DCs Vaccine	23	Transient increase in T2/FLAIR hyperintensity (n=3) Headaches (n=1)
Fadul et al. 2011 (113)	NCT	I	GBM	Irradiated tumor lysate DCs Vaccine	10	Neck pain (n=1)
Jie et al. 2012 (114)	NCT	II	GBM	Heat shocked tumor cells DCs Vaccine	13	None reported
Cho et al. 2012 (115)	NCT	II	GBM	Tumor lysate from surgical specimen DCs Vaccine	18	Post-op hemiplegia (n=1) Elevated ICP (n=1)
Ardon et al 2012 (116)	NCT	I	GBM	Tumor lysate from surgical specimen DCs Vaccine	77	Grade 4 status epilepticus (n=4) Grade 4 ischemic stroke (n=1) Grade 3/4 Grade 3/4 dementia (n=1) Seizures (n=5)
Akiyama et al. 2012 (72)	UMIN ID 000000914	I	AA, AO, GBM	Synthetic peptides (WT-1, HER2, MAGE-A3, MAGE-A1, and gp100) DCs Vaccine	9	None reported
Iwami et al. 2012 (74)	NCT	I	AA, AO, GBM	IL-13Rα2 peptide DCs Vaccine	8	None reported
Lasky et al. 2013 (117)	NCT00107185	I	AA, AO, GBM	Tumor lysate from surgical specimen DCs Vaccine	7	Headache (n=7)
Phuphanich et al. 2013 (77)	NCT	I	GBM	TAA epitopes synthetic peptides (HER2, TRP-2, gp100, MAGE-1, IL-13Rα2, and AIM-2) DCs Vaccine	21	None reported
Vik-Mo et al. 2013 (118)	NCT00846456	I/II	GBM	Transfection of mRNA from glioma stem cells DCs Vaccine	7	Seizures (n=1)
Prins et al. 2013 (119)	NCT00612001	I	AA, GBM	Peptide, Tumor Lysate GAAs (urviving, her-2/neu, gp100, and TRP-2) DCs Vaccine	34	Seizures (n=6) Photophobia (n=1) Vertigo/dizziness (n=2) Diplopia (n=1)
Hunn et al. 2015 (120)	NCT	I	GBM	Autologous tumor lysate previously exposed to TMZ DCs Vaccine	13	Grade 3 syncopal event (n=1) Post-op neurological deficit (n=1) Seizure (n=3) Headache (n=2)
Mitchell et al. 2015 (121)	NCT00639639	I/II	GBM	Transfected synthetic pp65 mRNA from CMV	12	None reported
Sakai et al. 2015 (73)	NCT	I	AA, AO, GBM	WT-1 antigen and/or tumor lysate from surgical specimen DCs Vaccine	10	None reported
Inogés et al. 2017 (122)	NCT01006044	II	GBM	Tumor lysate from surgical specimen DCs Vaccine	31	None reported
Batich et al. 2017 (75)	NCT00639639	I	rGBM	Transfected synthetic pp65 mRNA from CMV admixed with GM-CSF DC Vaccine	11	None reported
Liau et al. 2018 (79)	NCT00045968	III	nGBM	autologous tumor lysate (DCVax®-L) DC Vaccine	232	Cerebral edema (0.9%) Seizures (0.6%)
Wen et al. 2019 (78)	NCT01280552	II	nGBM	ICT-107 (MAGE-1, AIM-2, HER2/neu, TRP-2, IL13Rα2) DC Vaccine	81	Grade 2 Headache (2.5%) Grade 2 convulsions (6.3%) Grade 2 partial seizures (6.3%) Grade 2 hemiparesis (5%) Grade 3 Convulsions (5%)
Mitsuya et al. 2020 (123)	NCT01213407	II	nHGG	IL-12/IFN-γ DCs Vaccine	15	None reported
Wang et al. 2020 (124)	NCT02808416	I	nGBM, rGBM	DC Vaccine	5	None reported
Rudnick et al. 2020 (125)	NCT00576446	I	AA, AO, GBM	Tumor lysate from surgical specimen DCs Vaccine	28	Grade 1 dizziness Grade 1 speech difficulties Grade I aphasia
Ogino et al 2022 (83)	NCT02549833	I	LGG	GBM6-AD, lysate of an allogeneic glioblastoma stem cell line, with poly-ICLC	9	Headache (n=16) Dizziness (n=3) Seizure (n=1)
Hu et al. 2022 (82)	NCT02010606	I	nGBM, rGBM	Stem cell line lysate DCs Vaccine	36	None reported
Bota et al. 2022 (80)	NCT03400917	II	nGBM	Tumor lysate from surgical specimen DCs Vaccine	57	Headaches (n=21, 36.8%) Seizure (n=19, 33%) Focal weakness (n=16, 28.1%) Fall (n=10, 17.5%) Dizziness (n=10, 17.5%) Cerebral edema (n=9, 15.8%) Confused/forgetful (n=6, 10.5%)

-n, new diagnosis; -r, recurrent tumor; AA, anaplastic astrocytoma; AGG, anaplastic ganglioglioma; AO, anaplastic oligodendroglioma; AOA, anaplastic oligoastrocytoma; ATRT, atypical teratoid-rhabdoid tumor; CMV, cytomegalovirus; CNS, central nervous system; DCs, dendritic cells; DIPG, diffuse intrinsic pontine glioma; EPM, ependymoma; GAA, glioma associated antigen; GBM, glioblastoma; GM-CSF, granulocyte-macrophage colony-stimulating factor; HLA, human leukocyte antigen; HGG, high-grade glioma; ICP, intracranial pressure; IDH, isocitrate dehydrogenase; IFN, interferon; IL, interleukin; KLH, keyhole limpet hemocyanin; m, months; MB, medulloblastoma; MHC, major histocompatibility complex;; mRNA, messenger ribonucleic acid; Poly-ICLC, polyinosinic-polycytidylic acid stabilized by lysine and carboxymethylcellulose; PNET, primitive neuro-ectodermal tumor; PXA, pleomorphic xanthoastrocytoma; Td, tetanus diphtheria; TMZ, temozolomide; WT, Wilms tumor.

**Table 3:** Phase II/III trials of checkpoint inhibitors in glioblastoma.

Trial	NCT	Population	Trial design
Nivolumab CheckMate 548 (128)	NCT02667587	Newly diagnosed MGMT methylated GBM	Phase 3, randomized trial of standard of care RT + TMZ with or without nivolumab ( n =716)
Nivolumab CheckMate 498 (129)	NCT02617589	Newly diagnosed MGMT unmethylated GBM	Phase 3, randomized trial of RT + TMZ versus RT + nivolumab ( n=560)
Nivolumab (Ahluwalia et al.) (130)	NCT03452579	GBM, first relapse, dexamethasone dose ≤ 4 mg or equivalents	Phase 2, randomized trial of nivolumab 240 mg + bevacizumab standard dose 10 mg/kg (n = 45) or low dose 3 mg/kg IV (n = 45) every 2 weeks
Durvalumab (Reardon et al.) (1)	NCT02336165	Newly diagnosed MGMT unmethylated GBM	Phase 2, single-arm study of RT + durvalumab ( n=40)
CheckMate 143 (Reardon et al.) (131)	NCT02017717	GBM, first relapse, steroid dose < 10 mg prednisone equivalents	Phase 3, randomized trial of nivolumab 3 mg/kg (n = 184) or bevacizumab 10 mg/kg (n = 185) IV every 2 weeks
Pembrolizumab Nayak et al. (132)	NCT02337491	GBM, first or second relapse, dexamethasone dose ≤ 4 mg or equivalents	Phase 2, randomized trial of pembrolizumab (n = 50) or pembrolizumab + bevacizumab (n = 50)
Pembrolizumab KEYNOTE-028 (133)	NCT02054806	GBM cohort, recurrent, PD-L1 ≥ 1% by IHC, bevacizumab naïve	GBM cohort (n = 26) of basket study, phase 2 study of pembrolizumab 10 mg/kg every 2 weeks
Durvalumab (Reardon, et al.) (134)	NCT02336165	GBM, recurrent	Phase 2durvalumab (Cohort B, n = 30); durvalumab + bevacizumab 3 mg/kg every 2 weeks (Cohort B2, n = 33); durvalumab + bevacizumab 10 mg/kg every 2 weeks (Cohort B3, n = 33); bevacizumab refractory, durvalumab (Cohort C, n = 22)

Checkmate 548 evaluated the role of nivolumab in newly diagnosed glioblastoma in combination with standard of care (SOC) radiation and temozolomide therapy. The most frequent neurological adverse events in both arms were headache (Nivolumab + SOC, 9.3%; Placebo+ SOC, 5.9%) and dysgeusia (Nivolumab + SOC, 5.6%; Placebo+SOC, 4.2%), whereas the most frequent serious neurologic adverse event in both arms was reported as tumor flare (2.5%/1.4%) or pseudoprogression, which is a condition in which treatment related increased vascular permeability produces a transient increase in apparent tumor burden followed by tumor regression. This is a well described phenomenon which frequently occurs after chemoradiation in up to 50% of patients with newly diagnosed glioblastoma, making scan interpretation difficult (135). Pseudoprogression was evaluated in patients treated with temozolomide, radiation and nivolumab who had progression free survival of ≤6 months from the first nivolumab dose. Patients with stable follow-up scans ≥3-months following determination of preliminary progression and no worsening while remaining on treatment were considered as having pseudoprogression. Only 20 patients (5.6%) in the nivolumab + SOC arm were determined to have pseudoprogression per iRANO criteria. CheckMate 498 was a randomized phase III study investigating the efficacy of nivolumab and radiation compared with conventional chemoradiation in patients with newly diagnosed glioblastoma with negative MGMT promoter methylation. The trial did not meet its primary endpoint, i.e., SOC therapy was associated with superior overall survival compared with nivolumab and radiation (median overall survival, 14.9 vs. 13.4 months). Neurological adverse events again occurred in only 16.5% (grade 3/4, 1.8%) of patients in the nivolumab arm and 9.5% (grade 3/4, 0%) of patients treated on the SOC arm and were mostly headaches and dysgeusia. The grade 3 and 4 neurologic adverse event in the nivolumab arm consisted of cerebral edema, hemiparesis, and seizure. There were no serious neurologic adverse events in the control arm. In the relapsed setting, CheckMate 143, a phase III study of nivolumab versus bevacizumab also did not demonstrate a survival benefit of PD-! Inhibition versus bevacizumab. In the phase 1 portion of the study, anti-PD-1 monoclonal antibody nivolumab was given with or without the anti-CTLA-4 monoclonal antibody ipilimumab to patients with recurrent disease. That study showed that the toxicity profile in this population was consistent with the other trials. Cerebral edema, focal deficits and headaches were common and were mostly attributed to disease rather than treatment related toxicity. No new safety signals were identified. Importantly, there was no evidence of clinically significant neurotoxicity (131). While true checkpoint inhibitor related neurotoxicity was low across all checkpoint inhibitor glioblastoma trials, the main challenge has been rapid disease progression in the brain for the majority of patients who fail to respond to checkpoint inhibitors and the inability to distinguish actual disease progression from possible pseudoprogression leading to challenges in early response determination.

Based on the lack of efficacy with immunotherapy, there has been increased interest in evaluating combination of checkpoint inhibitors with other therapies including radiation therapy which may result in synergistic responses. Several ongoing trials are evaluating checkpoint blockade in combination with radiation therapy. For example, an ongoing trial is evaluating the benefit of adding radiation to CPIs. A phase 1 trial of recurrent GBM treated with atezolizumab, tociluzumab and stereotactic radiation (NCT04729959). Whether these and similar approaches lead to increased toxicity remains to be seen.

**Figure 1:**
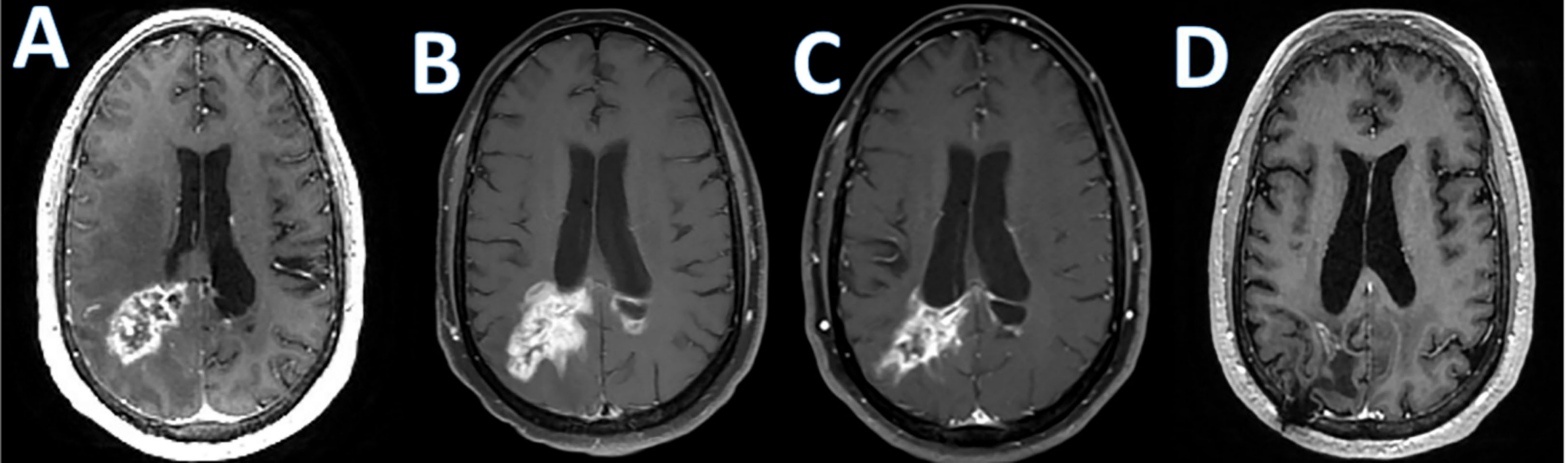
Checkpoint inhibitor associated pseudoprogression. T1 post-contrast enhanced MRI images in a patient with refractory primary central nervous system lymphoma treated with Pembrolizumab; **(A)**; baseline, **(B)**; increase in abnormal enhancement consistent with pseudo progression 9 weeks after starting pembrolizumab, **(C)**; minor response at 7 months; **(D)**; near complete response at 10 months.

Checkpoint blockade is also investigated as a treatment in PCNSL. A prospective study of nivolumab including 47 PCNSL patients (NCT02857426) demonstrated as serious adverse events edema or mass effect in 2/47 subjects and seizures in 4/47 subjects according to the available results posted on clinicaltrials.gov (136). Pembrolizumab has also been studied in relapsed PCNSL. The first results of a pembrolizumab phase II study demonstrated an overall response rate of 26%, a median PFS of 2.6 months. Most importantly, there was no significant CNS toxicity observed (137). However, similar to the experience with checkpoint inhibitors in other malignancies, pseudoprogression can be observed (**Figure 1**). There are several ongoing trials evaluating combinations of checkpoint inhibitors with other agents and whether this approach increases CNS toxicity remains to be seen.

Lastly, checkpoint inhibitors may also have a role in treatment refractory meningiomas. A single-arm, open-label phase 2 trial evaluating the efficacy of pembrolizumab, in 25 patients with recurrent and progressive grade 2 and 3 meningiomas met its primary endpoint and reported a median PFS of 7.6 months (90% CI: 3.4–12.9 months (138). The investigators reported only one serious neurologic adverse event which was grade-3 encephalopathy.

The current management of central neurologic complications from checkpoint inhibitors depends on the severity of neurologic deficits and follows established guidelines (139). An excessive inflammatory response can cause symptoms due to mass effect from vasogenic edema due to increased vascular permeability. This often necessitates treatment interruption and corticosteroids, and sometimes surgical debulking or bevacizumab. Steroids quickly reduce edema and diminish the associated immune response. The goal is to use the lowest effective dose and to attempt quick tapering if tolerated to minimize risk of long-term steroid toxicity and permit resumption of immunotherapy. Bevacizumab is sometimes used as a steroid sparing agent for patients who are steroid refractory.

CNS autoimmune toxicities such as encephalitis, aseptic meningitis, paraneoplastic disorders and demyelination due to checkpoint inhibitors which have very rarely been reported with the use of checkpoint inhibitors in other malignancies (140), have not been observed or reported with the use of checkpointing inhibitors in the published primary brain tumor trials.

## Oncolytic viruses

Viruses have been under evaluation as a promising treatment option in neuro-oncology for more than two decades. These viruses can either be non-lytic, in which case they are used to deliver therapeutic genes, or lytic, which means induction of tumor cell lysis and immune response. **Table 4** summarizes the completed oncolytic virus trials in patients with gliomas (143). Oncolytic viruses have recently emerged as a means to stimulate the innate and adaptive immune responses against both viral and tumor antigens and to reverse the immunosuppressive tumor microenvironment (144, 145), allowing for a more robust cytotoxic T cell-mediated antitumor response (146, 147). Such combination approaches have recently been explored in a phase 2 study of the oncolytic adenovirus immunotherapy DNX-2401 followed by the PD-1 inhibitor, pembrolizumab (CAPTIVE/KEYNOTE-192) and a similar approach has been explored in a recent study with PVSRIPO and pembrolizumab, (Luminos-101, NCT04479241).

**Table 4:** Surgical Window of Opportunity Checkpoint Inhibitor Trials.

Trial	NCT	Population	Trial design
Pembrolizumab (De Groot et al.) (141)	NCT02337686	GBM, first or second relapse, dexamethasone dose ≤ 2 mg or equivalents	Single arm surgical window of opportunity trial of neoadjuvant (up to 2 doses) and adjuvant pembrolizumab (n = 15)
Pembrolizumab (Cloughesy et al.) (127)	NCT02852655	GBM, first relapse	Randomized surgical window of opportunity trial of neoadjuvant + adjuvant pembrolizumab (n = 16) versus adjuvant pembrolizumab (n = 16)
Nivolumab (Schalper et al.) (142)	NCT02550249	GBM, 27 recurrent and 3 newly diagnosed	Single arm surgical window of opportunity trial of neoadjuvant and adjuvant nivolumab (n = 30)

Neurologic toxicity associated with intratumoral delivery of oncolytic viruses is common and usually related to increased vascular permeability, inflammation, vasogenic edema and mass effect and associated neurologic deficits (148). Toxicities have been primarily associated with viral replication and include fever, headache and malaise (143, 149), however, more serious complications such as encephalopathy, seizures, and cerebral edema were also observed (150). Despite theoretical concerns of off-receptor viral targeting or uncontrolled viral replication, studies have failed to demonstrate any evidence of neurovirulence thus far. Meningitis has rarely been observed in patients in whom the agent was inadvertently injected into CSF (150). The main toxicity in the published trials include peritumoral edema which is related directly to the intracerebral modes of administration (direct injection versus convection enhanced delivery), as well as the secondary immune response generated by the treatment. As a consequence, patients may experience localized neurologic deficits, such as weakness or aphasia; additionally, the risk of seizures is increased. In a study of 61 adult glioblastoma patients treated with intra-tumoral recombinant, live attenuated, nonpathogenic oncolytic virus containing the oral poliovirus Sabin type 1, PVS-RIPO, most patients experienced neurologic adverse events though the majority were not severe. Neurologic adverse events included: headache (52%), hemiparesis (50%), seizures (45%), dysphagia (28%), mental status change (25%), visual field deficits (19%), paresthesia (13%), abnormal gait (10%), dystonia (2%), and facial weakness (2%) (151). There were only three severe adverse events, including grade 4 cerebral edema (2%), grade 5 intracranial hemorrhage (2%) and grade 5 seizure (2%). For symptoms due to edema and mass effect, bevacizumab was used as supportive agent. Symptoms of viral malaise, and headache were managed supportively (151).

Management strategies include both symptom-directed treatment, such as for seizures, and control of the edema itself. While steroids are a commonly utilized treatment for edema, high doses of steroids can potentially suppress the immune response generated by oncolytic virotherapy, thereby reducing treatment efficacy. For refractory edema, bevacizumab, can be utilized as a steroid-sparing agent. Bevacizumab decreases edema by normalizing decreased vascular permeability. It has been used safely at least 2 weeks after completion of oncolytic virotherapy infusion (151). Other toxicities that have been rarely observed are intracerebral hemorrhage after tumor resection, or due to direct intratumoral injection, or catheter placement and removal as well as hydrocephalus.

**Figure 2:**
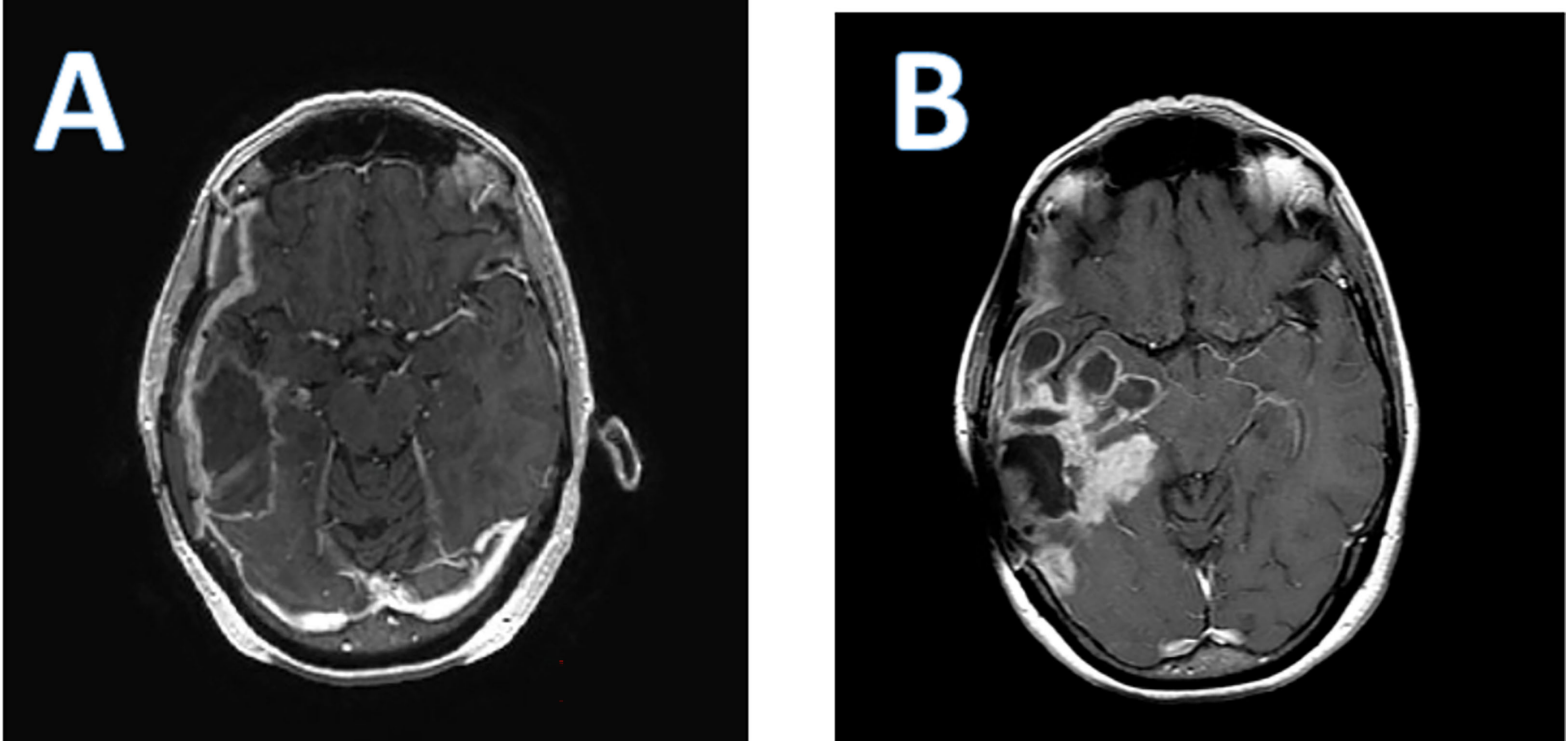
Axial T 1 post contrast enhanced brain MRI of a patient who received PVS-RIPO therapy. **(A)**; baseline; **(B)**; 4 months after PVS-RIPO, the MRI reveals cystic tissue degradation and parenchymal brain inflammation.

As is the case with all immunotherapies, a particular challenge has been the difficulty in distinguishing pseudoprogression due to the immunotherapeutic effect from true tumor progression and lack of efficacy. Post-treatment MRIs often demonstrate an increase in peritumoral edema, and an increase in lesion size with polycystic degeneration, also known as a “soap bubble” appearance (**Figure 2**).

## Discussion

Cancer immunotherapy is an exciting and emerging therapeutic field for extracranial malignancies with the promise of forward progress and marked improvement in patient outcomes in several solid tumor malignancies. Yet, in primary brain tumors, failures of later phase clinical trials evaluating immunotherapy treatments have highlighted the ongoing challenges of single agent immunotherapy in the treatment of brain cancer with ongoing and planned trials evaluating combinations of immunotherapeutics including approaches aimed at changing the immunosuppressive tumor microenvironment (152).

The application of immunotherapy to primary brain tumors has carried with it a substantial concern for immune-associated CNS neurologic complications. While serious immune mediated adverse events have been overall rare in primary brain tumor immunotherapy trials, the reporting of CNS immune adverse events has been inconsistent between trials and there are clear challenges in distinguishing immune mediated adverse events from disease progression, due to the overlapping presentations of immune-related toxicity, tumor pseudoprogression and true tumor progression. Similarly, imaging correlates of pseudoprogression overlap with tumor progression resulting in implementation of new radiographic diagnostic criteria to evaluate response in immunotherapy trials, termed iRANO (153). Key components of CNS toxicity concerns include the potential for unpredicted off-tumor, off-target cross-reactivity, or non-specific targeting and molecular mimicry.

Immunotherapy-associated CNS toxicity in primary brain tumors has not been studied systematically, is often under-recognized and underreported, and limited to case reports and case series of more severe events. As such, we reviewed the CNS complications associated with checkpoint inhibitors, oncolytic viruses, adoptive cell transfer/CAR T cell and vaccines for primary brain tumors to help clarify and understand the spectrum of immune-related neurological CNS toxicity.

Adoptive cell transfer/CAR T therapy associated neurological toxicity termed ‘‘tumor inflammation-associated neurotoxicity’ (TIAN) by Majzner et al., 2022 (21) is broken down into two major categories: 1) increased ICP due to inflammation-induced tissue edema and/or obstruction of CSF flow, and 2) primary dysfunction of brain or spinal cord structures due to inflammation. Of the published literature using CAR-T in primary brain tumors (**Table 5**), 3 of 7 clinical trials reported on the incidence of pseudoprogression with incidence 100% (24), 0% (25)and 25% (21)with four studies failing to report presence or absence of pseudoprogression. Likewise, the most frequent serious Grade II-IV CNS AEs reported across these published studies in a total of 54 participants were headache (5 of 7 studies), seizure (3 of 7 studies), and cerebral edema (3 of 7 studies), yet only one reported grade IV toxicity and only one reported neurologic DLT (**Table 5**). Using TIAN categories, the most common neurologic toxicity is related to category 2 primary dysfunction of brain structures resulting in headaches, seizures, and symptomatic cerebral edema. Management of TIAN varied with the use of corticosteroids, hypertonic saline, anti-cytokine agents (anakinra and tocilizumab). Notably, these studies highlighted the overall safety of varying routes of delivery (intracavitary, intraventricular and intravenous) and underscored the need for clear and timely management algorithms to mitigate potentially fatal treatment associated toxicity.

**Table 5:** Completed and Published Oncolytic Virus Trials for Primary Brain Tumors.

Agent	Study Population	Studies/ Phases	Sample Size
HSV 1716 (154–156)	Glioma (newly diagnosed / recurrent)	Phase I	33
DNX-2401 (157–162)	Relapsed GBM and DIPG	Phase I-II studies	156
G207 (163, 164)	Recurrent Glioma	Phase I-II studies	30
Adv-tk (165)	Newly diagnosed GBM	Phase II	48
Onyx-015 (166)	Recurrent glioma	Phase I	24
Toca 511 (167, 168)	Relapsed GBM	Phase I-III	244
PVSRIPO (151, 169)	Relapsed GBM	Phase I/Phase II	61/ 149
Reovirus (170)	Relapsed Glioma	Phase I	12
H1-Parvovirus (171)	Relapsed GBM	Phase I	18
NSC-CRAd-S-pk7 (150)	Newly diagnosed GBM	Phase I	12

Vaccine therapies have also been shown to be safe and well tolerated. From 2001-2020, over 23 published clinical trials encompassing 692 patients treated with a DC vaccine, showed only 10 Grade IV neurological AEs recorded including cerebral edema (n=2), seizures/status epilepticus (n=5), ischemic stroke (n=2) and Grade III/IV dementia (n=1). There were only two Grade III neurological AEs reported: grade III seizures (n=1) and syncopal event (n=1). Several trials reported seizures, headaches, transient post-operative neurological deficits, and dizziness without applying a grade to the events as specified by the National Cancer Institute Common Terminology Criteria for the Reporting of Adverse Events (NCI-CTCAE). Five trials had no reported neurological adverse events. Seven trials reported radiographic imaging changes with report of leukoencephalopathy (71), cerebral edema (71, 79, 96, 102, 106) and transient increase in T2/FLAIR hyperintensity (112) with three of the trials not assigning a CTCAE grade.

Combining the data from all 56 vaccine clinical trials encompassing peptide, heat shock protein and DC vaccines, the most frequently reported CNS adverse event was headache with a total of 187 incidents followed by seizure (n=78), cerebral edema (n=32), weakness/paresis (n=20), dizziness (n=17), fall (n=11), confusion (n=8), focal neurological deficits (n=6), aphasia/speech difficulty (n=5) and status epilepticus (n=5) as listed in **Table 6**. Among the adverse events that were assigned a CTCAE grade, status epilepticus was the most common Grade IV toxicity (n=5) followed by seizure (n=2), cerebral edema (n=2) and ischemic stroke (n=2). There was substantial variation in reporting of adverse events among the publications. Notably, a few of the reported adverse events may not have been related to vaccination administration, such as reporting of post-operative neurological deficits or dementia. Despite this variation and lack of uniform reporting of events, most adverse events were mild, suggesting that vaccine therapy has a reasonable safety profile.

**Table 6:** Summary of All CNS Adverse Events across 56 Vaccine Clinical Trials.

Grade						
Adverse Events	1 n (%)	2 n (%)	3 n (%)	4 n (%)	Unassigned n (%)	Total n (%)
Headache	33(1.74%)	10(0.53%)	20(1.06%)		130(6.87%)	187(9.8%)
Seizure	6(0.32%)	24 (1.27%)	20(1.06%)	2(0.11%)	26(1.37%)	78(4.12%)
Cerebral edema		1(0.05%)	6(0.32%)	4(0.21%)	21(1.11%)	32(1.69%)
Weakness/paresis	3(0.16%)	12(0.63%)	5(0.26%)			20(1.06%)
Dizziness	9(0.48%)	2(0.11%)			6(0.32%)	17(0.9%)
Fall	6(0.32%)	1(0.05%)	2(0.11%)	2(0.11%)		11(0.58%)
Confusion	2(0.11%)	3(0.16%)	1(0.05%)		2(0.11%)	8(0.42%)
Focal neurological deficits					6(0.32%)	6(0.32%)
Aphasia/Speech Difficulty	2(0.11%)				3(0.16%)	5(0.26%)
Status epilepticus				5(0.26%)		5(0.26%)
Back/Neck Pain			2(0.11%)		1(0.05%)	3(0.16%)
Transient T2/FLAIR hyperintensity					3(0.16%)	3(0.16%)
Chemical meningitis					2(0.11%)	2(0.11%)
Ischemic stroke				2(0.11%)		2(0.11%)
Paresthesia/Abnormal sensation					2(0.11%)	2(0.11%)
Photophobia					2(0.11%)	2(0.11%)
Post-operative neurological deficit					2(0.11%)	2(0.11%)
Dementia				1(0.05%)		1(0.05%)
Diplopia					1(0.05%)	1(0.05%)
Dysguesia					1(0.05%)	1(0.05%)
Increased ICP					1(0.05%)	1(0.05%)
Leukoencephalopathy	1(0.05%)					1(0.05%)
Syncope			1(0.05%)			1(0.05%)

The total number of participants across 56 vaccine clinical trials was 1,892. The percentage (%) is equal to the total number of events divided by the total number of participants across the 56 clinical trials.

The spectrum of checkpoint inhibitor induced neurologic autoimmunity has been extensively reviewed in the literature. In studies of checkpoint inhibitors for primary brain tumor patients specifically, however, CNS complications such as vasculitis, meningoencephalitis, retinopathy, posterior reversible encephalopathy, cerebellar degeneration, autoimmune encephalitis, and progression of multiple sclerosis have either not been observed at all or only very rarely been recorded (84). The main observed neurologic adverse events are largely neurologic deficits and headaches due to inflammatory brain changes, edema and mass effect and were rarely severe.

The main challenge has been rapid disease progression in the brain for the majority of patients unresponsive to checkpoint inhibitors and the difficulty in distinguishing actual disease progression from possible pseudoprogression leading to challenges in early response determination. In Checkmate 548 which has strictly applied iRANO criteria, only 5.6% of patients were determined to as having confirmed pseudoprogression (128) and it is likely that most of the neurologic adverse events reported across checkpoint inhibitor trials are in fact due to underlying disease progression rather than immune mediated effects.

As concerns oncolytic viruses, acute neurologic complications can often be more directly linked to the intratumoral administration of these agents, of which some are procedural complications. But the determination of secondary immune response generated by the treatment is equally challenging as with the other immunotherapeutics.

As the use of combinatorial treatment options including multi-agent checkpoint inhibitors, oncolytic viruses, adoptive cell transfer/CAR T cell and multi-antigen targeted vaccines for primary brain tumors (96) continues to grow, it is critical to define and report the unique CNS complications associated with such treatments to help guide accurate diagnosis and appropriate management to limit morbidity and improve or prevent the reduction of patients’ quality of life. Prospective clinical trials evaluating clinical and laboratory biomarkers that can help stratify or identify patients that may be more prone for neurological complications as well as trials specifically aimed at the diagnosis and treatment of CNS-associated immunotherapy complications in primary brain tumors is needed.

## Author contributions

Study design: MM, JD. Acquisition of data: MM, JD. Interpretation of the data: MM, JD. Drafting of the initial manuscript: MM, JD. Review and revision of the manuscript: MM, JD. Approval of the final manuscript: MM, JD. All authors contributed to the article and approved the submitted version.

## References

[1] Reardon DA Kaley TJ Dietrich J Clarke JL Dunn G Lim M. Phase II study to evaluate safety and efficacy of MEDI4736 (durvalumab) + radiotherapy in patients with newly diagnosed unmethylated MGMT glioblastoma (new unmeth GBM). J Clin Oncol (2019) 37:2032–2. doi: 10.1200/JCO.2019.37.15_suppl.2032

[2] Louis DN Perry A Wesseling P Brat DJ Cree IA Figarella-Branger D. The 2021 WHO classification of tumors of the central nervous system: a summary. Neuro Oncol (2021) 23:1231–51. doi: 10.1093/neuonc/noab106 PMC8328013 34185076

[3] Miller KD Ostrom QT Kruchko C Patil N Tihan T Cioffi G. Brain and other central nervous system tumor statistics, 2021. CA Cancer J Clin (2021) 71:381–406. doi: 10.3322/caac.21693 34427324

[4] Weller M Cloughesy T Perry JR Wick W. Standards of care for treatment of recurrent glioblastoma–are we there yet? Neuro Oncol (2013) 15:4–27. doi: 10.1093/neuonc/nos273 23136223 PMC3534423

[5] Hodi FS O’Day SJ McDermott DF Weber RW Sosman JA Haanen JB. Improved survival with ipilimumab in patients with metastatic melanoma. N Engl J Med (2010) 363:711–23. doi: 10.1056/NEJMoa1003466 PMC3549297 20525992

[6] Brahmer J Reckamp KL Baas P Crino L Eberhardt WE Poddubskaya E. Nivolumab versus docetaxel in advanced squamous-cell non-Small-Cell lung cancer. N Engl J Med (2015) 373:123–35. doi: 10.1056/NEJMoa1504627 PMC4681400 26028407

[7] Neelapu SS Locke FL Bartlett NL Lekakis LJ Miklos DB Jacobson CA. Axicabtagene ciloleucel CAR T-cell therapy in refractory Large b-cell lymphoma. N Engl J Med (2017) 377:2531–44. doi: 10.1056/NEJMoa1707447 PMC5882485 29226797

[8] Di Giacomo AM Valente M Cerase A Lofiego MF Piazzini F Calabro L. Immunotherapy of brain metastases: breaking a “dogma”. J Exp Clin Cancer Res (2019) 38:419. doi: 10.1186/s13046-019-1426-2 31623643 PMC6798349

[9] Burton LB Eskian M Guidon AC Reynolds KL. A review of neurotoxicities associated with immunotherapy and a framework for evaluation. Neurooncol Adv (2021) 3:v108–20. doi: 10.1093/noajnl/vdab107 PMC8633791 34859238

[10] Hong M Clubb JD Chen YY. Engineering CAR-T cells for next-generation cancer therapy. Cancer Cell (2020) 38:473–88. doi: 10.1016/j.ccell.2020.07.005 32735779

[11] Sermer D Brentjens R. CAR T-cell therapy: Full speed ahead. Hematol Oncol (2019) 37 Suppl 1:95–100. doi: 10.1002/hon.2591 31187533

[12] Hawkins ER D’Souza RR Klampatsa A. Armored CAR T-cells: The next chapter in T-cell cancer immunotherapy. Biologics (2021) 15:95–105. doi: 10.2147/BTT.S291768 33883875 PMC8053711

[13] Styczyński J. A brief history of CAR-T cells: from laboratory to the bedside. Acta Haematologica Polonica (2020) 51:2–5. doi: 10.2478/ahp-2020-0002

[14] Lin YJ Mashouf LA Lim M. CAR T cell therapy in primary brain tumors: Current investigations and the future. Front Immunol (2022) 13:817296. doi: 10.3389/fimmu.2022.817296 35265074 PMC8899093

[15] Gust J Ponce R Liles WC Garden GA Turtle CJ. Cytokines in CAR T cell-associated neurotoxicity. Front Immunol (2020) 11:577027. doi: 10.3389/fimmu.2020.577027 33391257 PMC7772425

[16] Zhao WH Liu J Wang BY Chen YX Cao XM Yang Y. A phase 1, open-label study of LCAR-B38M, a chimeric antigen receptor T cell therapy directed against b cell maturation antigen, in patients with relapsed or refractory multiple myeloma. J Hematol Oncol (2018) 11:141. doi: 10.1186/s13045-018-0681-6 30572922 PMC6302465

[17] Brammer JE Braunstein Z Katapadi A Porter K Biersmith M Guha A. Early toxicity and clinical outcomes after chimeric antigen receptor T-cell (CAR-T) therapy for lymphoma. J Immunother Cancer (2021) 9(8):e002303. doi: 10.1136/jitc-2020-002303 34429331 PMC8386216

[18] Rubin DB Danish HH Ali AB Li K LaRose S Monk AD. Neurological toxicities associated with chimeric antigen receptor T-cell therapy. Brain (2019) 142:1334–48. doi: 10.1093/brain/awz053 30891590

[19] Gust J Hay KA Hanafi LA Li D Myerson D Gonzalez-Cuyar LF. Endothelial activation and blood-brain barrier disruption in neurotoxicity after adoptive immunotherapy with CD19 CAR-T cells. Cancer Discovery (2017) 7:1404–19. doi: 10.1158/2159-8290.CD-17-0698 PMC5718945 29025771

[20] Gust J Finney OC Li D Brakke HM Hicks RM Futrell RB. Glial injury in neurotoxicity after pediatric CD19-directed chimeric antigen receptor T cell therapy. Ann Neurol (2019) 86:42–54. doi: 10.1002/ana.25502 31074527 PMC9375054

[21] Majzner RG Ramakrishna S Yeom KW Patel S Chinnasamy H Schultz LM. GD2-CAR T cell therapy for H3K27M-mutated diffuse midline gliomas. Nature (2022) 603:934–41. doi: 10.1038/s41586-022-04489-4 PMC8967714 35130560

[22] Mintz A Gibo DM Slagle-Webb B Christensen ND Debinski W. IL-13Ralpha2 is a glioma-restricted receptor for interleukin-13. Neoplasia (2002) 4:388–99. doi: 10.1038/sj.neo.7900234 PMC1564118 12192597

[23] Tu M Wange W Cai L Zhu P Gao Z Zheng W. IL-13 receptor alpha2 stimulates human glioma cell growth and metastasis through the Src/PI3K/Akt/mTOR signaling pathway. Tumour Biol (2016) 37:14701–9. doi: 10.1007/s13277-016-5346-x 27623944

[24] Brown CE Badie B Barish ME Weng L Ostberg JR Chang WC. Bioactivity and safety of IL13Ralpha2-redirected chimeric antigen receptor CD8+ T cells in patients with recurrent glioblastoma. Clin Cancer Res (2015) 21:4062–72. doi: 10.1158/1078-0432.CCR-15-0428 PMC4632968 26059190

[25] Brown CE Alizadeh D Starr R Weng L Wagner JR Naranjo A. Regression of glioblastoma after chimeric antigen receptor T-cell therapy. N Engl J Med (2016) 375:2561–9. doi: 10.1056/NEJMoa1610497 PMC5390684 28029927

[26] Gan HK Cvrljevic AN Johns TG. The epidermal growth factor receptor variant III (EGFRvIII): where wild things are altered. FEBS J (2013) 280:5350–70. doi: 10.1111/febs.12393 23777544

[27] O’Rourke DM Nasrallah MP Desai A Melenhorst JJ Mansfield K Morrissette JJD. A single dose of peripherally infused EGFRvIII-directed CAR T cells mediates antigen loss and induces adaptive resistance in patients with recurrent glioblastoma. Sci Transl Med (2017) 9(399):eaaa0984. doi: 10.1126/scitranslmed.aaa0984 28724573 PMC5762203

[28] Goff SL Morgan RA Yang JC Sherry RM Robbins PF Restifo NP. Pilot trial of adoptive transfer of chimeric antigen receptor-transduced T cells targeting EGFRvIII in patients with glioblastoma. J Immunother (2019) 42:126–35. doi: 10.1097/CJI.0000000000000260 PMC6691897 30882547

[29] Tang X Wang Y Huang J Zhang Z Liu F Xu J. Administration of B7-H3 targeted chimeric antigen receptor-T cells induce regression of glioblastoma. Signal Transduct Target Ther (2021) 6:125. doi: 10.1038/s41392-021-00505-7 33767145 PMC7994554

[30] Zhang JG Kruse CA Driggers L Hoa N Wisoff J Allen JC. Tumor antigen precursor protein profiles of adult and pediatric brain tumors identify potential targets for immunotherapy. J Neurooncol (2008) 88:65–76. doi: 10.1007/s11060-008-9534-4 18259692 PMC4005736

[31] Ahmed N Brawley V Hegde M Bielamowicz K Kalra M Landi D. HER2-specific chimeric antigen receptor-modified virus-specific T cells for progressive glioblastoma: A phase 1 dose-escalation trial. JAMA Oncol (2017) 3:1094–101. doi: 10.1001/jamaoncol.2017.0184 PMC5747970 28426845

[32] Frey NV Porter DL. Cytokine release syndrome with novel therapeutics for acute lymphoblastic leukemia. Hematol Am Soc Hematol Educ Program (2016) 2016:567–72. doi: 10.1182/asheducation-2016.1.567 PMC6142489 27913530

[33] Thakar MS Kearl TJ Malarkannan S. Controlling cytokine release syndrome to harness the full potential of CAR-based cellular therapy. Front Oncol (2019) 9:1529. doi: 10.3389/fonc.2019.01529 32076597 PMC7006459

[34] Grupp SA Kalos M Barrett D Aplenc R Porter DL Rheingold SR. Chimeric antigen receptor-modified T cells for acute lymphoid leukemia. N Engl J Med (2013) 368:1509–18. doi: 10.1056/NEJMoa1215134 PMC4058440 23527958

[35] Teachey DT Rheingold SR Maude SL Zugmaier G Barrett DM Seif AE. Cytokine release syndrome after blinatumomab treatment related to abnormal macrophage activation and ameliorated with cytokine-directed therapy. Blood (2013) 121:5154–7. doi: 10.1182/blood-2013-02-485623 PMC4123427 23678006

[36] Caimi PF Pacheco Sanchez G Sharma A Otegbeye F Ahmed N Rojas P. Prophylactic tocilizumab prior to anti-CD19 CAR-T cell therapy for non-Hodgkin lymphoma. Front Immunol (2021) 12:745320. doi: 10.3389/fimmu.2021.745320 34712233 PMC8546323

[37] Neelapu SS Tummala S Kebriaei P Wierda W Gutierrez C Locke FL. Chimeric antigen receptor T-cell therapy - assessment and management of toxicities. Nat Rev Clin Oncol (2018) 15:47–62. doi: 10.1038/nrclinonc.2017.148 28925994 PMC6733403

[38] Chen F Teachey DT Pequignot E Frey N Porter D Maude SL. Measuring IL-6 and sIL-6R in serum from patients treated with tocilizumab and/or siltuximab following CAR T cell therapy. J Immunol Methods (2016) 434:1–8. doi: 10.1016/j.jim.2016.03.005 27049586 PMC5490247

[39] Giavridis T van der Stegen SJC Eyquem J Hamieh M Piersigilli A Sadelain M. CAR T cell-induced cytokine release syndrome is mediated by macrophages and abated by IL-1 blockade. Nat Med (2018) 24:731–8. doi: 10.1038/s41591-018-0041-7 PMC6410714 29808005

[40] Sterner RM Sakemura R Cox MJ Yang N Khadka RH Forsman CL. GM-CSF inhibition reduces cytokine release syndrome and neuroinflammation but enhances CAR-T cell function in xenografts. Blood (2019) 133:697–709. doi: 10.1182/blood-2018-10-881722 30463995 PMC6376281

[41] Richardson PG Palomo M Kernan NA Hildebrandt GC Chao N Carreras E. The importance of endothelial protection: The emerging role of defibrotide in reversing endothelial injury and its sequelae. Bone Marrow Transplant (2021) 56:2889–96. doi: 10.1038/s41409-021-01383-x PMC8477726 34584241

[42] Jones BS Lamb LS Goldman F Di Stasi A. Improving the safety of cell therapy products by suicide gene transfer. Front Pharmacol (2014) 5:254. doi: 10.3389/fphar.2014.00254 25505885 PMC4245885

[43] Zhou X Dotti G Krance RA Martinez CA Naik S Kamble RT. Inducible caspase-9 suicide gene controls adverse effects from alloreplete T cells after haploidentical stem cell transplantation. Blood (2015) 125:4103–13. doi: 10.1182/blood-2015-02-628354 PMC4481597 25977584

[44] Gargett T Brown MP. The inducible caspase-9 suicide gene system as a “safety switch” to limit on-target, off-tumor toxicities of chimeric antigen receptor T cells. Front Pharmacol (2014) 5:235. doi: 10.3389/fphar.2014.00235 25389405 PMC4211380

[45] Brudno JN Kochenderfer JN. Recent advances in CAR T-cell toxicity: Mechanisms, manifestations and management. Blood Rev (2019) 34:45–55. doi: 10.1016/j.blre.2018.11.002 30528964 PMC6628697

[46] McGranahan T Therkelsen KE Ahmad S Nagpal S. Current state of immunotherapy for treatment of glioblastoma. Curr Treat Options Oncol (2019) 20:24. doi: 10.1007/s11864-019-0619-4 30790064 PMC6394457

[47] Weller M Roth P Preusser M Wick W Reardon DA Platten M. Vaccine-based immunotherapeutic approaches to gliomas and beyond. Nat Rev Neurol (2017) 13:363–74. doi: 10.1038/nrneurol.2017.64 28497804

[48] Medikonda R Dunn G Rahman M Fecci P Lim M. A review of glioblastoma immunotherapy. J Neurooncol (2021) 151:41–53. doi: 10.1007/s11060-020-03448-1 32253714

[49] Bigner DD Pitts OM Wikstrand CJ. Induction of lethal experimental allergic encephalomyelitis in nonhuman primates and guinea pigs with human glioblastoma multiforme tissue. J Neurosurg (1981) 55:32–42. doi: 10.3171/jns.1981.55.1.0032 6165811

[50] Frederico SC Hancock JC Brettschneider EES Ratnam NM Gilbert MR Terabe M. Making a cold tumor hot: The role of vaccines in the treatment of glioblastoma. Front Oncol (2021) 11:672508. doi: 10.3389/fonc.2021.672508 34041034 PMC8141615

[51] Weller M Butowski N Tran DD Recht LD Lim M Hirte H. Rindopepimut with temozolomide for patients with newly diagnosed, EGFRvIII-expressing glioblastoma (ACT IV): a randomised, double-blind, international phase 3 trial. Lancet Oncol (2017) 18:1373–85. doi: 10.1016/S1470-2045(17)30517-X 28844499

[52] Narita Y Arakawa Y Yamasaki F Nishikawa R Aoki T Kanamori M. A randomized, double-blind, phase III trial of personalized peptide vaccination for recurrent glioblastoma. Neuro Oncol (2019) 21:348–59. doi: 10.1093/neuonc/noy200 PMC6380422 30500939

[53] Liau LM Ashkan K Brem S Campian JL Trusheim JE Iwamoto FM. Association of Autologous Tumor Lysate-Loaded Dendritic Cell Vaccination With Extension of Survival Among Patients With Newly Diagnosed and Recurrent Glioblastoma: A Phase 3 Prospective Externally Controlled Cohort Trial. JAMA Oncol (2022). 10.1001/jamaoncol.2022.5370 PMC9673026 36394838

[54] Bijker MS Melief CJ Offringa R van der Burg SH. Design and development of synthetic peptide vaccines: past, present and future. Expert Rev Vaccines (2007) 6:591–603. doi: 10.1586/14760584.6.4.591 17669012

[55] Kong Z Wang Y Ma W. Vaccination in the immunotherapy of glioblastoma. Hum Vaccin Immunother (2018) 14:255–68. doi: 10.1080/21645515.2017.1388481 PMC5806656 29087782

[56] Lim M Xia Y Bettegowda C Weller M. Current state of immunotherapy for glioblastoma. Nat Rev Clin Oncol (2018) 15:422–42. doi: 10.1038/s41571-018-0003-5 29643471

[57] Reardon DA Desjardins A Vredenburgh JJ O’Rourke DM Tran DD Fink KL. Rindopepimut with bevacizumab for patients with relapsed EGFRvIII-expressing glioblastoma (ReACT): Results of a double-blind randomized phase II trial. Clin Cancer Res (2020) 26:1586–94. doi: 10.1158/1078-0432.CCR-18-1140 32034072

[58] Cuoco JA Benko MJ Busch CM Rogers CM Prickett JT Marvin EA. Vaccine-based immunotherapeutics for the treatment of glioblastoma: Advances, challenges, and future perspectives. World Neurosurg (2018) 120:302–15. doi: 10.1016/j.wneu.2018.08.202 30196171

[59] Thompson E Landi D Thompson E Lipp E Balajonda B Herndon J. Peptide vaccine directed to cmv Pp65 for treatment of recurrent malignant glioma and medulloblastoma in children and young adults: Preliminary results of a phase I trial. Neuro-Oncology (2020) 22:ii37–7. doi: 10.1093/neuonc/noaa215.155

[60] Platten M Bunse L Wick W. Emerging targets for anticancer vaccination: IDH. ESMO Open (2021) 6:100214. doi: 10.1016/j.esmoop.2021.100214 34271312 PMC8287141

[61] Platten M Bunse L Wick A Bunse T Le Cornet L Harting I. A vaccine targeting mutant IDH1 in newly diagnosed glioma. Nature (2021) 592:463–8. doi: 10.1038/s41586-021-03363-z PMC8046668 33762734

[62] Suginobe N Nakamura M Takanashi Y Ban H Gotoh M. Mechanism of action of DSP-7888 (adegramotide/nelatimotide) emulsion, a peptide-based therapeutic cancer vaccine with the potential to turn up the heat on non-immunoreactive tumors. Clin Transl Oncol (2022) 25(2):396–407. doi: 10.1007/s12094-022-02946-0 PMC9510518 36138335

[63] Fenstermaker RA Ciesielski MJ Qiu J Yang N Frank CL Lee KP. Clinical study of a survivin long peptide vaccine (SurVaxM) in patients with recurrent malignant glioma. Cancer Immunol Immunother (2016) 65(11):1339–1352. doi: 10.1007/s00262-016-1890-x PMC5069322 27576783

[64] Pollack IF Jakacki RI Butterfield LH Hamilton RL Panigrahy A Potter DM. Antigen-specific immune responses and clinical outcome after vaccination with glioma-associated antigen peptides and polyinosinic-polycytidylic acid stabilized by lysine and carboxymethylcellulose in children with newly diagnosed malignant brainstem and nonbrainstem gliomas. J Clin Oncol (2014) 32:2050–8. doi: 10.1200/JCO.2013.54.0526 PMC4067943 24888813

[65] Migliorini D Dutoit V Allard M Grandjean Hallez N Marinari E Widmer V. Phase I/II trial testing safety and immunogenicity of the multipeptide IMA950/poly-ICLC vaccine in newly diagnosed adult malignant astrocytoma patients. Neuro Oncol (2019) 21:923–33. doi: 10.1093/neuonc/noz040 PMC6620642 30753611

[66] Rampling R Peoples S Mulholland PJ James A Al-Salihi O Twelves CJ. A cancer research UK first time in human phase I trial of IMA950 (Novel multipeptide therapeutic vaccine) in patients with newly diagnosed glioblastoma. Clin Cancer Res (2016) 22:4776–85. doi: 10.1158/1078-0432.CCR-16-0506 PMC5026298 27225692

[67] Herrada AA Rojas-Colonelli N Gonzalez-Figueroa P Roco J Oyarce C Ligtenberg MA. Harnessing DNA-induced immune responses for improving cancer vaccines. Hum Vaccin Immunother (2012) 8:1682–93. doi: 10.4161/hv.22345 PMC3601143 23111166

[68] Reardon DA Brem S Desai AS Bagley SJ Kurz SC Fuente MIDL. Intramuscular (IM) INO-5401 + INO-9012 with electroporation (EP) in combination with cemiplimab (REGN2810) in newly diagnosed glioblastoma. J Clin Oncol (2022) 40:2004–4. doi: 10.1200/JCO.2022.40.16_suppl.2004

[69] Reardon DA Mitchell DA. The development of dendritic cell vaccine-based immunotherapies for glioblastoma. Semin Immunopathol (2017) 39:225–39. doi: 10.1007/s00281-016-0616-7 28138787

[70] Srivastava S Jackson C Kim T Choi J Lim M. A characterization of dendritic cells and their role in immunotherapy in glioblastoma: From preclinical studies to clinical trials. Cancers (Basel) (2019) 11(4):537. doi: 10.3390/cancers11040537 30991681 PMC6521200

[71] Sampson JH Heimberger AB Archer GE Aldape KD Friedman AH Friedman HS. Immunologic escape after prolonged progression-free survival with epidermal growth factor receptor variant III peptide vaccination in patients with newly diagnosed glioblastoma. J Clin Oncol (2010) 28:4722–9. doi: 10.1200/JCO.2010.28.6963 PMC3020702 20921459

[72] Akiyama Y Oshita C Kume A Iizuka A Miyata H Komiyama M. Alpha-type-1 polarized dendritic cell-based vaccination in recurrent high-grade glioma: a phase I clinical trial. BMC Cancer (2012) 12:623. doi: 10.1186/1471-2407-12-623 23270484 PMC3541167

[73] Sakai K Shimodaira S Maejima S Udagawa N Sano K Higuchi Y. Dendritic cell-based immunotherapy targeting wilms’ tumor 1 in patients with recurrent malignant glioma. J Neurosurg (2015) 123:989–97. doi: 10.3171/2015.1.JNS141554 26252465

[74] Iwami K Shimato S Ohno M Okada H Nakahara N Sato Y. Peptide-pulsed dendritic cell vaccination targeting interleukin-13 receptor alpha2 chain in recurrent malignant glioma patients with HLA-A*24/A*02 allele. Cytotherapy (2012) 14:733–42. doi: 10.3109/14653249.2012.666633 22424217

[75] Batich KA Reap EA Archer GE Sanchez-Perez L Nair SK Schmittling RJ. Long-term survival in glioblastoma with cytomegalovirus pp65-targeted vaccination. Clin Cancer Res (2017) 23:1898–909. doi: 10.1158/1078-0432.CCR-16-2057 PMC5559300 28411277

[76] Batich KA Mitchell DA Healy P Herndon JE 2nd Sampson JH. Once, twice, three times a finding: Reproducibility of dendritic cell vaccine trials targeting cytomegalovirus in glioblastoma. Clin Cancer Res (2020) 26:5297–303. doi: 10.1158/1078-0432.CCR-20-1082 PMC9832384 32719000

[77] Phuphanich S Wheeler CJ Rudnick JD Mazer M Wang H Nuno MA. Phase I trial of a multi-epitope-pulsed dendritic cell vaccine for patients with newly diagnosed glioblastoma. Cancer Immunol Immunother (2013) 62:125–35. doi: 10.1007/s00262-012-1319-0 PMC3541928 22847020

[78] Wen PY Reardon DA Armstrong TS Phuphanich S Aiken RD Landolfi JC. A randomized double-blind placebo-controlled phase II trial of dendritic cell vaccine ICT-107 in newly diagnosed patients with glioblastoma. Clin Cancer Res (2019) 25:5799–807. doi: 10.1158/1078-0432.CCR-19-0261 PMC8132111 31320597

[79] Liau LM Ashkan K Tran DD Campian JL Trusheim JE Cobbs CS. First results on survival from a large phase 3 clinical trial of an autologous dendritic cell vaccine in newly diagnosed glioblastoma. J Transl Med (2018) 16:142. doi: 10.1186/s12967-018-1507-6 29843811 PMC5975654

[80] Bota DA Taylor TH Piccioni DE Duma CM LaRocca RV Kesari S. Phase 2 study of AV-GBM-1 (a tumor-initiating cell targeted dendritic cell vaccine) in newly diagnosed glioblastoma patients: safety and efficacy assessment. J Exp Clin Cancer Res (2022) 41:344. doi: 10.1186/s13046-022-02552-6 36517865 PMC9749349

[81] Lathia JD Mack SC Mulkearns-Hubert EE Valentim CL Rich JN. Cancer stem cells in glioblastoma. Genes Dev (2015) 29:1203–17. doi: 10.1101/gad.261982.115 PMC4495393 26109046

[82] Hu JL Omofoye OA Rudnick JD Kim S Tighiouart M Phuphanich S. A phase I study of autologous dendritic cell vaccine pulsed with allogeneic stem-like cell line lysate in patients with newly diagnosed or recurrent glioblastoma. Clin Cancer Res (2022) 28:689–96. doi: 10.1158/1078-0432.CCR-21-2867 34862245

[83] Ogino H Taylor JW Nejo T Gibson D Watchmaker PB Okada K. Randomized trial of neoadjuvant vaccination with tumor-cell lysate induces T cell response in low-grade gliomas. J Clin Invest (2022) 132(3):e151239. doi: 10.1172/JCI151239 34882581 PMC8803342

[84] Cuzzubbo S Carpentier AF. Neurological adverse events of immune checkpoint blockade: from pathophysiology to treatment. Curr Opin Neurol (2022) 35:814–22. doi: 10.1097/WCO.0000000000001113 36226705

[85] Black KL Ciacci JD Ammirati M Selch MT Becker DP. Clinical trial of serratia marcescens extract and radiation therapy in patients with malignant astrocytoma. J Clin Oncol (1993) 11:1746–50. doi: 10.1200/JCO.1993.11.9.1746 8394880

[86] Sampson JH Archer GE Mitchell DA Heimberger AB Herndon JE 2nd Lally-Goss D. An epidermal growth factor receptor variant III-targeted vaccine is safe and immunogenic in patients with glioblastoma multiforme. Mol Cancer Ther (2009) 8:2773–9. doi: 10.1158/1535-7163.MCT-09-0124 PMC2991139 19825799

[87] Yu JS Liu G Ying H Yong WH Black KL Wheeler CJ. Vaccination with tumor lysate-pulsed dendritic cells elicits antigen-specific, cytotoxic T-cells in patients with malignant glioma. Cancer Res (2004) 64:4973–9. doi: 10.1158/0008-5472.CAN-03-3505 15256471

[88] Sampson JH Aldape KD Archer GE Coan A Desjardins A Friedman AH. Greater chemotherapy-induced lymphopenia enhances tumor-specific immune responses that eliminate EGFRvIII-expressing tumor cells in patients with glioblastoma. Neuro Oncol (2011) 13:324–33. doi: 10.1093/neuonc/noq157 PMC3064599 21149254

[89] Schuster J Lai RK Recht LD Reardon DA Paleologos NA Groves MD. Multicenter trial of rindopepimut (CDX-110) in newly diagnosed glioblastoma: the ACT III study. Neuro Oncol (2015) 17:854–61. doi: 10.1093/neuonc/nou348 PMC4483122 25586468

[90] Reardon DA Desjardins A Schuster J Tran DD Fink KL Nabors LB. IMCT-08ReACT: Long-term survival from a randomized phase ii study of rindopepimut (CDX-110) plus bevacizumab in relapsed glioblastoma. Neuro-Oncology (2015) 17:v109–9. doi: 10.1093/neuonc/nov218.08

[91] Crane CA Han SJ Ahn B Oehlke J Kivett V Fedoroff A. Individual patient-specific immunity against high-grade glioma after vaccination with autologous tumor derived peptides bound to the 96 KD chaperone protein. Clin Cancer Res (2013) 19:205–14. doi: 10.1158/1078-0432.CCR-11-3358 22872572

[92] Bloch O Crane CA Fuks Y Kaur R Aghi MK Berger MS. Heat-shock protein peptide complex-96 vaccination for recurrent glioblastoma: a phase II, single-arm trial. Neuro Oncol (2014) 16:274–9. doi: 10.1093/neuonc/not203 PMC3895386 24335700

[93] Fenstermaker RA Ciesielski MJ Qiu J Yang N Frank CL Lee KP. Clinical study of a survivin long peptide vaccine (SurVaxM) in patients with recurrent malignant glioma. Cancer Immunol Immunother (2016) 65:1339–52. doi: 10.1007/s00262-016-1890-x PMC5069322 27576783

[94] Rosenfeld MR Chamberlain MC Grossman SA Peereboom DM Lesser GJ Batchelor TT. A multi-institution phase II study of poly-ICLC and radiotherapy with concurrent and adjuvant temozolomide in adults with newly diagnosed glioblastoma. Neuro Oncol (2010) 12:1071–7. doi: 10.1093/neuonc/noq071 PMC3018929 20615924

[95] Wheeler CJ Black KL Liu G Mazer M Zhang XX Pepkowitz S. Vaccination elicits correlated immune and clinical responses in glioblastoma multiforme patients. Cancer Res (2008) 68:5955–64. doi: 10.1158/0008-5472.CAN-07-5973 18632651

[96] Hilf N Kuttruff-Coqui S Frenzel K Bukur V Stevanovic S Gouttefangeas C. Actively personalized vaccination trial for newly diagnosed glioblastoma. Nature (2019) 565:240–5. doi: 10.1038/s41586-018-0810-y 30568303

[97] Keskin DB Anandappa AJ Sun J Tirosh I Mathewson ND Li S. Neoantigen vaccine generates intratumoral T cell responses in phase ib glioblastoma trial. Nature (2019) 565:234–9. doi: 10.1038/s41586-018-0792-9 PMC6546179 30568305

[98] Ishikawa E Tsuboi K Yamamoto T Muroi A Takano S Enomoto T. Clinical trial of autologous formalin-fixed tumor vaccine for glioblastoma multiforme patients. Cancer Sci (2007) 98:1226–33. doi: 10.1111/j.1349-7006.2007.00518.x PMC11158799 17517052

[99] Ishikawa E Muragaki Y Yamamoto T Maruyama T Tsuboi K Ikuta S. Phase I/IIa trial of fractionated radiotherapy, temozolomide, and autologous formalin-fixed tumor vaccine for newly diagnosed glioblastoma. J Neurosurg (2014) 121:543–53. doi: 10.3171/2014.5.JNS132392 24995786

[100] Kikuchi T Akasaki Y Irie M Homma S Abe T Ohno T. Results of a phase I clinical trial of vaccination of glioma patients with fusions of dendritic and glioma cells. Cancer Immunol Immunother (2001) 50:337–44. doi: 10.1007/s002620100205 PMC11032998 11676393

[101] Yamanaka R Abe T Yajima N Tsuchiya N Homma J Kobayashi T. Vaccination of recurrent glioma patients with tumour lysate-pulsed dendritic cells elicits immune responses: results of a clinical phase I/II trial. Br J Cancer (2003) 89:1172–9. doi: 10.1038/sj.bjc.6601268 PMC2394324 14520441

[102] Rutkowski S De Vleeschouwer S Kaempgen E Wolff JE Kuhl J Demaerel P. Surgery and adjuvant dendritic cell-based tumour vaccination for patients with relapsed malignant glioma, a feasibility study. Br J Cancer (2004) 91:1656–62. doi: 10.1038/sj.bjc.6602195 PMC2409960 15477864

[103] Okada H Lieberman FS Walter KA Lunsford LD Kondziolka DS Bejjani GK. Autologous glioma cell vaccine admixed with interleukin-4 gene transfected fibroblasts in the treatment of patients with malignant gliomas. J Transl Med (2007) 5:67. doi: 10.1186/1479-5876-5-67 18093335 PMC2254376

[104] Caruso DA Orme LM Amor GM Neale AM Radcliff FJ Downie P. Results of a phase I study utilizing monocyte-derived dendritic cells pulsed with tumor RNA in children with stage 4 neuroblastoma. Cancer (2005) 103:1280–91. doi: 10.1002/cncr.20911 15693021

[105] Liau LM Prins RM Kiertscher SM Odesa SK Kremen TJ Giovannone AJ. Dendritic cell vaccination in glioblastoma patients induces systemic and intracranial T-cell responses modulated by the local central nervous system tumor microenvironment. Clin Cancer Res (2005) 11:5515–25. doi: 10.1158/1078-0432.CCR-05-0464 16061868

[106] De Vleeschouwer S Fieuws S Rutkowski S Van Calenbergh F Van Loon J Goffin J. Postoperative adjuvant dendritic cell-based immunotherapy in patients with relapsed glioblastoma multiforme. Clin Cancer Res (2008) 14:3098–104. doi: 10.1158/1078-0432.CCR-07-4875 18483377

[107] Walker DG Laherty R Tomlinson FH Chuah T Schmidt C. Results of a phase I dendritic cell vaccine trial for malignant astrocytoma: potential interaction with adjuvant chemotherapy. J Clin Neurosci (2008) 15:114–21. doi: 10.1016/j.jocn.2007.08.007 18083572

[108] Ardon H Van Gool S Lopes IS Maes W Sciot R Wilms G. Integration of autologous dendritic cell-based immunotherapy in the primary treatment for patients with newly diagnosed glioblastoma multiforme: a pilot study. J Neurooncol (2010) 99:261–72. doi: 10.1007/s11060-010-0131-y 20146084

[109] Ardon H De Vleeschouwer S Van Calenbergh F Claes L Kramm CM Rutkowski S. Adjuvant dendritic cell-based tumour vaccination for children with malignant brain tumours. Pediatr Blood Cancer (2010) 54:519–25. doi: 10.1002/pbc.22319 19852061

[110] Chang AL Miska J Wainwright DA Dey M Rivetta CV Yu D. CCL2 produced by the glioma microenvironment is essential for the recruitment of regulatory T cells and myeloid-derived suppressor cells. Cancer Res (2016) 76:5671–82. doi: 10.1158/0008-5472.CAN-16-0144 PMC5050119 27530322

[111] Okada H Kalinski P Ueda R Hoji A Kohanbash G Donegan TE. Induction of CD8+ T-cell responses against novel glioma-associated antigen peptides and clinical activity by vaccinations with alpha-type 1 polarized dendritic cells and polyinosinic-polycytidylic acid stabilized by lysine and carboxymethylcellulose in patients with recurrent malignant glioma. J Clin Oncol (2011) 29:330–6. doi: 10.1200/JCO.2010.30.7744 PMC3056467 21149657

[112] Prins RM Soto H Konkankit V Odesa SK Eskin A Yong WH. Gene expression profile correlates with T-cell infiltration and relative survival in glioblastoma patients vaccinated with dendritic cell immunotherapy. Clin Cancer Res (2011) 17:1603–15. doi: 10.1158/1078-0432.CCR-10-2563 PMC3071163 21135147

[113] Fadul CE Fisher JL Hampton TH Lallana EC Li Z Gui J. Immune response in patients with newly diagnosed glioblastoma multiforme treated with intranodal autologous tumor lysate-dendritic cell vaccination after radiation chemotherapy. J Immunother (2011) 34:382–9. doi: 10.1097/CJI.0b013e318215e300 PMC3766324 21499132

[114] Jie X Hua L Jiang W Feng F Feng G Hua Z. Clinical application of a dendritic cell vaccine raised against heat-shocked glioblastoma. Cell Biochem Biophys (2012) 62:91–9. doi: 10.1007/s12013-011-9265-6 21909820

[115] Cho DY Yang WK Lee HC Hsu DM Lin HL Lin SZ. Adjuvant immunotherapy with whole-cell lysate dendritic cells vaccine for glioblastoma multiforme: a phase II clinical trial. World Neurosurg (2012) 77:736–44. doi: 10.1016/j.wneu.2011.08.020 22120301

[116] Ardon H Van Gool SW Verschuere T Maes W Fieuws S Sciot R. Integration of autologous dendritic cell-based immunotherapy in the standard of care treatment for patients with newly diagnosed glioblastoma: results of the HGG-2006 phase I/II trial. Cancer Immunol Immunother (2012) 61:2033–44. doi: 10.1007/s00262-012-1261-1 PMC11028710 22527250

[117] Lasky JL 3rd Panosyan EH Plant A Davidson T Yong WH Prins RM. Autologous tumor lysate-pulsed dendritic cell immunotherapy for pediatric patients with newly diagnosed or recurrent high-grade gliomas. Anticancer Res (2013) 33:2047–56. PMC4018463 23645755

[118] Vik-Mo EO Nyakas M Mikkelsen BV Moe MC Due-Tonnesen P Suso EM. Therapeutic vaccination against autologous cancer stem cells with mRNA-transfected dendritic cells in patients with glioblastoma. Cancer Immunol Immunother (2013) 62:1499–509. doi: 10.1007/s00262-013-1453-3 PMC3755221 23817721

[119] Prins RM Wang X Soto H Young E Lisiero DN Fong B. Comparison of glioma-associated antigen peptide-loaded versus autologous tumor lysate-loaded dendritic cell vaccination in malignant glioma patients. J Immunother (2013) 36:152–7. doi: 10.1097/CJI.0b013e3182811ae4 PMC3568250 23377664

[120] Hunn MK Bauer E Wood CE Gasser O Dzhelali M Ancelet LR. Dendritic cell vaccination combined with temozolomide retreatment: results of a phase I trial in patients with recurrent glioblastoma multiforme. J Neurooncol (2015) 121:319–29. doi: 10.1007/s11060-014-1635-7 25366363

[121] Mitchell DA Batich KA Gunn MD Huang MN Sanchez-Perez L Nair SK. Tetanus toxoid and CCL3 improve dendritic cell vaccines in mice and glioblastoma patients. Nature (2015) 519:366–9. doi: 10.1038/nature14320 PMC4510871 25762141

[122] Inoges S Tejada S de Cerio AL Gallego Perez-Larraya J Espinos J Idoate MA. A phase II trial of autologous dendritic cell vaccination and radiochemotherapy following fluorescence-guided surgery in newly diagnosed glioblastoma patients. J Transl Med (2017) 15:104. doi: 10.1186/s12967-017-1202-z 28499389 PMC5427614

[123] Mitsuya K Akiyama Y Iizuka A Miyata H Deguchi S Hayashi N. Alpha-type-1 polarized dendritic cell-based vaccination in newly diagnosed high-grade glioma: A phase II clinical trial. Anticancer Res (2020) 40:6473–84. doi: 10.21873/anticanres.14669 33109586

[124] Wang QT Nie Y Sun SN Lin T Han RJ Jiang J. Tumor-associated antigen-based personalized dendritic cell vaccine in solid tumor patients. Cancer Immunol Immunother (2020) 69:1375–87. doi: 10.1007/s00262-020-02496-w PMC11027674 32078016

[125] Rudnick JD Sarmiento JM Uy B Nuno M Wheeler CJ Mazer MJ. A phase I trial of surgical resection with gliadel wafer placement followed by vaccination with dendritic cells pulsed with tumor lysate for patients with malignant glioma. J Clin Neurosci (2020) 74:187–93. doi: 10.1016/j.jocn.2020.03.006 32169363

[126] Bouffet E Larouche V Campbell BB Merico D de Borja R Aronson M. Immune checkpoint inhibition for hypermutant glioblastoma multiforme resulting from germline biallelic mismatch repair deficiency. J Clin Oncol (2016) 34:2206–11. doi: 10.1200/JCO.2016.66.6552 27001570

[127] Cloughesy TF Mochizuki AY Orpilla JR Hugo W Lee AH Davidson TB. Neoadjuvant anti-PD-1 immunotherapy promotes a survival benefit with intratumoral and systemic immune responses in recurrent glioblastoma. Nat Med (2019) 25:477–86. doi: 10.1038/s41591-018-0337-7 PMC6408961 30742122

[128] Lim M Weller M Idbaih A Steinbach J Finocchiaro G Raval RR. Phase III trial of chemoradiotherapy with temozolomide plus nivolumab or placebo for newly diagnosed glioblastoma with methylated MGMT promoter. Neuro Oncol (2022) 24:1935–49. doi: 10.1093/neuonc/noac116 PMC9629431 35511454

[129] Omuro A Brandes AA Carpentier AF Idbaih A Reardon DA Cloughesy T. Radiotherapy combined with nivolumab or temozolomide for newly diagnosed glioblastoma with unmethylated MGMT promoter: An international randomized phase 3 trial. Neuro Oncol (2022) 25(1):123–34. doi: 10.1093/neuonc/noac099 PMC9825306 35419607

[130] Ahluwalia MS Rauf Y Li H Wen PY Peereboom DM Reardon DA. Randomized phase 2 study of nivolumab (nivo) plus either standard or reduced dose bevacizumab (bev) in recurrent glioblastoma (rGBM). J Clin Oncol (2021) 39:2015–5. doi: 10.1200/JCO.2021.39.15_suppl.2015

[131] Reardon DA Brandes AA Omuro A Mulholland P Lim M Wick A. Effect of nivolumab vs bevacizumab in patients with recurrent glioblastoma: The CheckMate 143 phase 3 randomized clinical trial. JAMA Oncol (2020) 6:1003–10. doi: 10.1001/jamaoncol.2020.1024 PMC7243167 32437507

[132] Nayak L Molinaro AM Peters K Clarke JL Jordan JT de Groot J. And biomarker study of pembrolizumab plus bevacizumab versus pembrolizumab alone for patients with recurrent glioblastoma. Clin Cancer Res (2021) 27:1048–57. doi: 10.1158/1078-0432.CCR-20-2500 PMC8284901 33199490

[133] Reardon DA Kim TM Frenel JS Simonelli M Lopez J Subramaniam DS. Treatment with pembrolizumab in programmed death ligand 1-positive recurrent glioblastoma: Results from the multicohort phase 1 KEYNOTE-028 trial. Cancer (2021) 127:1620–9. doi: 10.1002/cncr.33378 33496357

[134] Reardon DA Kaley TJ Dietrich J Clarke JL Dunn GP Lim M. Phase 2 study to evaluate safety and efficacy of MEDI4736 (durvalumab [DUR]) in glioblastoma (GBM) patients: An update. J Clin Oncol (2017) 35:2042–2. doi: 10.1200/JCO.2017.35.15_suppl.2042

[135] Hagiwara A Schlossman J Shabani S Raymond C Tatekawa H Abrey LE. Incidence, molecular characteristics, and imaging features of “clinically-defined pseudoprogression” in newly diagnosed glioblastoma treated with chemoradiation. J Neurooncol (2022) 159:509–18. doi: 10.1007/s11060-022-04088-3 PMC13030911 35842871

[136] Nayak L Iwamoto FM Ferreri AJ Santoro A Singer S Batlevi C. Checkmate 647: A phase 2, open-label study of nivolumab in Relapsed/Refractory primary central nervous system lymphoma or Relapsed/Refractory primary testicular lymphoma. Hematological Oncol (2017) 35:420–1. doi: 10.1002/hon.2440_2

[137] Hoang-Xuan K Houot R Soussain C Blonski M Schmitt A Delwail V. First results of the acsé pembrolizumab phase II in the primary CNS lymphoma (PCNSL) cohort. Blood (2020) 136:15–6. doi: 10.1182/blood-2020-141773

[138] Brastianos PK Kim AE Giobbie-Hurder A Lee EQ Wang N Eichler AF. Phase 2 study of pembrolizumab in patients with recurrent and residual high-grade meningiomas. Nat Commun (2022) 13:1325. doi: 10.1038/s41467-022-29052-7 35289329 PMC8921328

[139] Schneider BJ Naidoo J Santomasso BD Lacchetti C Adkins S Anadkat M. Management of immune-related adverse events in patients treated with immune checkpoint inhibitor therapy: ASCO guideline update. J Clin Oncol (2021) 39:4073–126. doi: 10.1200/JCO.21.01440 34724392

[140] Zhao Z Zhang C Zhou L Dong P Shi L. Immune checkpoint inhibitors and neurotoxicity. Curr Neuropharmacol (2021) 19:1246–63. doi: 10.2174/1570159X19666201230151224 PMC8719293 33380303

[141] de Groot J Penas-Prado M Alfaro-Munoz K Hunter K Pei BL O’Brien B. Window-of-opportunity clinical trial of pembrolizumab in patients with recurrent glioblastoma reveals predominance of immune-suppressive macrophages. Neuro Oncol (2020) 22:539–49. doi: 10.1093/neuonc/noz185 PMC7158647 31755915

[142] Schalper KA Rodriguez-Ruiz ME Diez-Valle R Lopez-Janeiro A Porciuncula A Idoate MA. Neoadjuvant nivolumab modifies the tumor immune microenvironment in resectable glioblastoma. Nat Med (2019) 25:470–6. doi: 10.1038/s41591-018-0339-5 30742120

[143] Suryawanshi YR Schulze AJ. Oncolytic viruses for malignant glioma: On the verge of success? Viruses (2021) 13(7):1294. doi: 10.3390/v13071294 34372501 PMC8310195

[144] Pearl TM Markert JM Cassady KA Ghonime MG. Oncolytic virus-based cytokine expression to improve immune activity in brain and solid tumors. Mol Ther Oncolytics (2019) 13:14–21. doi: 10.1016/j.omto.2019.03.001 30997392 PMC6453942

[145] Gujar S Pol JG Kim Y Lee PW Kroemer G. Antitumor benefits of antiviral immunity: An underappreciated aspect of oncolytic virotherapies. Trends Immunol (2018) 39:209–21. doi: 10.1016/j.it.2017.11.006 29275092

[146] Kim Y Konda P Murphy JP Paulo JA Gygi SP Gujar S. Immune checkpoint blockade augments changes within oncolytic virus-induced cancer MHC-I peptidome, creating novel antitumor CD8 T cell reactivities. Mol Cell Proteomics (2022) 21:100182. doi: 10.1016/j.mcpro.2021.100182 34922008 PMC8864471

[147] Asija S Chatterjee A Yadav S Chekuri G Karulkar A Jaiswal AK. Combinatorial approaches to effective therapy in glioblastoma (GBM): Current status and what the future holds. Int Rev Immunol (2022) 41:582–605. doi: 10.1080/08830185.2022.2101647 35938932

[148] Marelli G Howells A Lemoine NR Wang Y. Oncolytic viral therapy and the immune system: A double-edged sword against cancer. Front Immunol (2018) 9. doi: 10.3389/fimmu.2018.00866 PMC5932159 29755464

[149] Wang JL Scheitler KM Wenger NM Elder JB. Viral therapies for glioblastoma and high-grade gliomas in adults: a systematic review. Neurosurgical Focus FOC (2021) 50:E2. doi: 10.3171/2020.11.FOCUS20854 33524943

[150] Fares J Ahmed AU Ulasov IV Sonabend AM Miska J Lee-Chang C. Neural stem cell delivery of an oncolytic adenovirus in newly diagnosed malignant glioma: a first-in-human, phase 1, dose-escalation trial. Lancet Oncol (2021) 22:1103–14. doi: 10.1016/S1470-2045(21)00245-X PMC8328944 34214495

[151] Desjardins A Gromeier M Herndon JE Beaubier 2N Bolognesi DP Friedman AH. Bigner, recurrent glioblastoma treated with recombinant poliovirus. N Engl J Med (2018) 379:150–61. doi: 10.1056/NEJMoa1716435 PMC6065102 29943666

[152] Franson A McClellan BL Varela ML Comba A Syed MF Banerjee K. Development of immunotherapy for high-grade gliomas: Overcoming the immunosuppressive tumor microenvironment. Front Med (Lausanne) (2022) 9:966458. doi: 10.3389/fmed.2022.966458 36186781 PMC9515652

[153] Okada H Weller M Huang R Finocchiaro G Gilbert MR Wick W. Immunotherapy response assessment in neuro-oncology: a report of the RANO working group. Lancet Oncol (2015) 16:e534–42. doi: 10.1016/S1470-2045(15)00088-1 PMC4638131 26545842

[154] Papanastassiou V Rampling R Fraser M Petty R Hadley D Nicoll J. The potential for efficacy of the modified (ICP 34.5(-)) herpes simplex virus HSV1716 following intratumoural injection into human malignant glioma: a proof of principle study. Gene Ther (2002) 9:398–406. doi: 10.1038/sj.gt.3301664 11960316

[155] Harrow S Papanastassiou V Harland J Mabbs R Petty R Fraser M. HSV1716 injection into the brain adjacent to tumour following surgical resection of high-grade glioma: safety data and long-term survival. Gene Ther (2004) 11:1648–58. doi: 10.1038/sj.gt.3302289 15334111

[156] Rampling R Cruickshank G Papanastassiou V Nicoll J Hadley D Brennan D. Toxicity evaluation of replication-competent herpes simplex virus (ICP 34.5 null mutant 1716) in patients with recurrent malignant glioma. Gene Ther (2000) 7:859–66. doi: 10.1038/sj.gt.3301184 10845724

[157] Gallego Perez-Larraya J Garcia-Moure M Labiano S Patino-Garcia A Dobbs J Gonzalez-Huarriz M. Oncolytic DNX-2401 virus for pediatric diffuse intrinsic pontine glioma. N Engl J Med (2022) 386:2471–81. doi: 10.1056/NEJMoa2202028 35767439

[158] Philbrick B Adamson DC. DNX-2401: an investigational drug for the treatment of recurrent glioblastoma. Expert Opin Investig Drugs (2019) 28:1041–9. doi: 10.1080/13543784.2019.1694000 31726894

[159] Tejada S Diez-Valle R Dominguez PD Patino-Garcia A Gonzalez-Huarriz M Fueyo J. DNX-2401, an oncolytic virus, for the treatment of newly diagnosed diffuse intrinsic pontine gliomas: A case report. Front Oncol (2018) 8:61. doi: 10.3389/fonc.2018.00061 29594041 PMC5858123

[160] The oncolytic adenovirus DNX-2401 has antitumor activity in glioblastoma. Cancer Discov (2018) 8(4):382. doi: 10.1158/2159-8290.CD-RW2018-031 29475886

[161] Lang FF Conrad C Gomez-Manzano C Yung WKA Sawaya R Weinberg JS. Phase I study of DNX-2401 (Delta-24-RGD) oncolytic adenovirus: Replication and immunotherapeutic effects in recurrent malignant glioma. J Clin Oncol (2018) 36:1419–27. doi: 10.1200/JCO.2017.75.8219 PMC6075856 29432077

[162] Tejada S Alonso M Patino A Fueyo J Gomez-Manzano C Diez-Valle R. Phase I trial of DNX-2401 for diffuse intrinsic pontine glioma newly diagnosed in pediatric patients. Neurosurgery (2018) 83:1050–6. doi: 10.1093/neuros/nyx507 29088386

[163] Markert JM Razdan SN Kuo HC Cantor A Knoll A Karrasch M. A phase 1 trial of oncolytic HSV-1, G207, given in combination with radiation for recurrent GBM demonstrates safety and radiographic responses. Mol Ther (2014) 22:1048–55. doi: 10.1038/mt.2014.22 PMC4015243 24572293

[164] Markert JM Medlock MD Rabkin SD Gillespie GY Todo T Hunter WD. Conditionally replicating herpes simplex virus mutant, G207 for the treatment of malignant glioma: results of a phase I trial. Gene Ther (2000) 7:867–74. doi: 10.1038/sj.gt.3301205 10845725

[165] Wheeler LA Manzanera AG Bell SD Cavaliere R McGregor JM Grecula JC. Phase II multicenter study of gene-mediated cytotoxic immunotherapy as adjuvant to surgical resection for newly diagnosed malignant glioma. Neuro Oncol (2016) 18:1137–45. doi: 10.1093/neuonc/now002 PMC4933478 26843484

[166] Chiocca EA Abbed KM Tatter S Louis DN Hochberg FH Barker F. A phase I open-label, dose-escalation, multi-institutional trial of injection with an E1B-attenuated adenovirus, ONYX-015, into the peritumoral region of recurrent malignant gliomas, in the adjuvant setting. Mol Ther (2004) 10:958–66. doi: 10.1016/j.ymthe.2004.07.021 15509513

[167] Cloughesy TF Petrecca K Walbert T Butowski N Salacz M Perry J. Effect of vocimagene amiretrorepvec in combination with flucytosine vs standard of care on survival following tumor resection in patients with recurrent high-grade glioma: A randomized clinical trial. JAMA Oncol (2020) 6:1939–46. doi: 10.1001/jamaoncol.2020.3161 PMC7596685 33119048

[168] Cloughesy TF Landolfi J Hogan DJ Bloomfield S Carter B Chen CC. Phase 1 trial of vocimagene amiretrorepvec and 5-fluorocytosine for recurrent high-grade glioma. Sci Transl Med (2016) 8:341ra75. doi: 10.1126/scitranslmed.aad9784 PMC6707068 27252174

[169] Desjardins A Gromeier M Friedman H Landi D Friedman A Ashley DM. Immu-26. safety and efficacy of pvsripo in recurrent glioblastoma: Long-term follow-up and initial multicenter results. Neuro-Oncology (2021) 23(Supplement_6):vi97–7. doi: 10.1093/neuonc/noab196.385

[170] Forsyth P Roldan G George D Wallace C Palmer CA Morris D. A phase I trial of intratumoral administration of reovirus in patients with histologically confirmed recurrent malignant gliomas. Mol Ther (2008) 16:627–32. doi: 10.1038/sj.mt.6300403 18253152

[171] Geletneky K Hajda J Angelova AL Leuchs B Capper D Bartsch AJ. Oncolytic h-1 parvovirus shows safety and signs of immunogenic activity in a first phase I/IIa glioblastoma trial. Mol Ther (2017) 25:2620–34. doi: 10.1016/j.ymthe.2017.08.016 PMC5768665 28967558

